# Disruption of the HIF-1 pathway in individuals with Ollier disease and Maffucci syndrome

**DOI:** 10.1371/journal.pgen.1010504

**Published:** 2022-12-08

**Authors:** Sarah R. Poll, Renan Martin, Elizabeth Wohler, Elizabeth S. Partan, Elizabeth Walek, Shaima Salman, Daniel Groepper, Lisa Kratz, Mirlene Cernach, Reynaldo Jesus-Garcia, Chad Haldeman-Englert, Yoon Jae Choi, Carol D. Morris, Bernard Cohen, Julie Hoover-Fong, David Valle, Gregg L. Semenza, Nara L. M. Sobreira

**Affiliations:** 1 McKusick-Nathans Department of Genetic Medicine, Johns Hopkins University School of Medicine, Baltimore, Maryland, United States of America; 2 Department of Pediatrics, Southern Illinois University School of Medicine, Springfield, Illinois, United States of America; 3 Universidade Metropolitana de Santos, Santos, São Paulo, Brazil; 4 Department of Orthopedics-Oncology, Universidade Federal de São Paulo, São Paulo, Brazil; 5 Mission Fullerton Genetics Center, Asheville, North Carolina, United States of America; 6 Department of Neurology, University of California, Irvine, California, United States of America; 7 Department of Orthopedic Surgery, Johns Hopkins School of Medicine, Baltimore, Maryland, United States of America; 8 Department of Oncology, Johns Hopkins School of Medicine, Baltimore, Maryland, United States of America; 9 Department of Dermatology, Johns Hopkins School of Medicine, Baltimore, Maryland, Untied States of America; Broad Institute, UNITED STATES

## Abstract

Ollier disease (OD) and Maffucci Syndrome (MS) are rare disorders characterized by multiple enchondromas, commonly causing bone deformities, limb length discrepancies, and pathological fractures. MS is distinguished from OD by the development of vascular anomalies. Both disorders are cancer predisposition syndromes with malignancies developing in ~50% of the individuals with OD or MS. Somatic gain-of-function variants in *IDH1* and *IDH2* have been described in the enchondromas, vascular anomalies and chondrosarcomas of approximately 80% of the individuals with OD and MS. To date, however, no investigation of germline causative variants for these diseases has been comprehensively performed. To search for germline causative variants, we performed whole exome sequencing or whole genome sequencing of blood or saliva DNA in 94 unrelated probands (68 trios). We found that 7 had rare germline missense variants in *HIF1A*, 6 had rare germline missense variants in *VHL*, and 3 had *IDH1* variants including 2 with mosaic *IDH1*-p.Arg132His variant. A burden analysis using 94 probands assigned as cases and 2,054 unrelated individuals presenting no OD- or MS-related features as controls, found that variants in *HIF1A*, *VHL*, and *IDH1* were all significantly enriched in cases compared to controls. To further investigate the role of HIF-1 pathway in the pathogenesis of OD and MS, we performed RNA sequencing of fibroblasts from 4 probands with OD or MS at normoxia and at hypoxia. When cultured in hypoxic conditions, both proband and control cells showed altered expression of a subset of HIF-1 regulated genes. However, the set of differentially expressed genes in proband fibroblasts included a significantly reduced number of HIF-1 regulated genes compared to controls. Our findings suggest that germline or early post-zygotic variants identified in *HIF1A*, *VHL*, and *IDH1* in probands with OD and MS underlie the development of the phenotypic abnormalities in a subset of individuals with OD and MS, but extensive functional studies are needed to further confirm it.

## Author summary

Ollier disease (OD) and Maffucci Syndrome (MS) are rare disorders characterized by multiple enchondromas, commonly causing bone deformities, limb length discrepancies, and pathological fractures. MS is distinguished from OD by the development of vascular anomalies. Both disorders are cancer predisposition syndromes with malignancies developing in ~50% of the individuals with OD or MS. Somatic gain-of-function variants in *IDH1* and *IDH2* have been described in the enchondromas, vascular anomalies and chondrosarcomas of approximately 80% of the individuals with OD and MS. To date, however, no investigation of germline causative variants for these diseases has been comprehensively performed. To search for germline causative variants, we performed whole exome sequencing or whole genome sequencing of blood or saliva DNA in 94 unrelated probands. We found that rare germline missense variants in *HIF1A*, *VHL*, *IDH1*. Our burden analysis found that variants in *HIF1A*, *VHL*, and *IDH1* were all significantly enriched in cases compared to controls. Our findings suggest that germline or early post-zygotic variants identified in *HIF1A*, *VHL*, and *IDH1* in probands with OD and MS underlie the development of the phenotypic abnormalities in a subset of individuals with OD and MS, but extensive functional studies are needed to further confirm it.

## Introduction

Ollier Disease (OD) and Maffucci Syndrome (MS) are phenotypically related disorders characterized by the presence of 3 or more enchondromas that usually develop during childhood. In addition, individuals with MS develop vascular anomalies in early adolescence that mostly affect the extremities. Recently, El Abiad at al. (2020) confirmed that both disorders are cancer predisposition syndromes [1]. Chondrosarcoma is the most prevalent cancer in both OD and MS with an overall incidence of approximately 30% [1]. There is also an increased risk for intracranial malignancies, specifically those of glial origin, and gonadal malignancies. Individuals with MS also have an increased incidence of vascular malignancies [1]. The enchondromas and vascular anomalies characteristic of OD and MS can affect any region of the body, but are most likely to develop in the appendicular skeleton where they cause pain, swelling, deformity, limitation in joint mobility, scoliosis, limb-length discrepancy and gait disturbances [1,2]. Pathological fractures, facial asymmetry and cranial nerve palsies can also occur [3].

The molecular bases of OD and MS are poorly understood; there is no evidence of familial recurrence, and associated germline variants have not been investigated. Somatic, heterozygous, gain-of-function (GoF) variants in *IDH1* (p.Arg132His, p.Arg132Cys, and p.Arg132Ser) and *IDH2* (p.Arg172Ser) occur in the enchondromas, vascular anomalies and chondrosarcomas of approximately 80% of the individuals with OD and MS but this is not consistent even in different enchondromas isolated from the same individual [4,5]. Moreover, several affected individuals do not have somatic *IDH1* or *IDH2* variants despite an intensive search [4,5].

**Fig 1:**
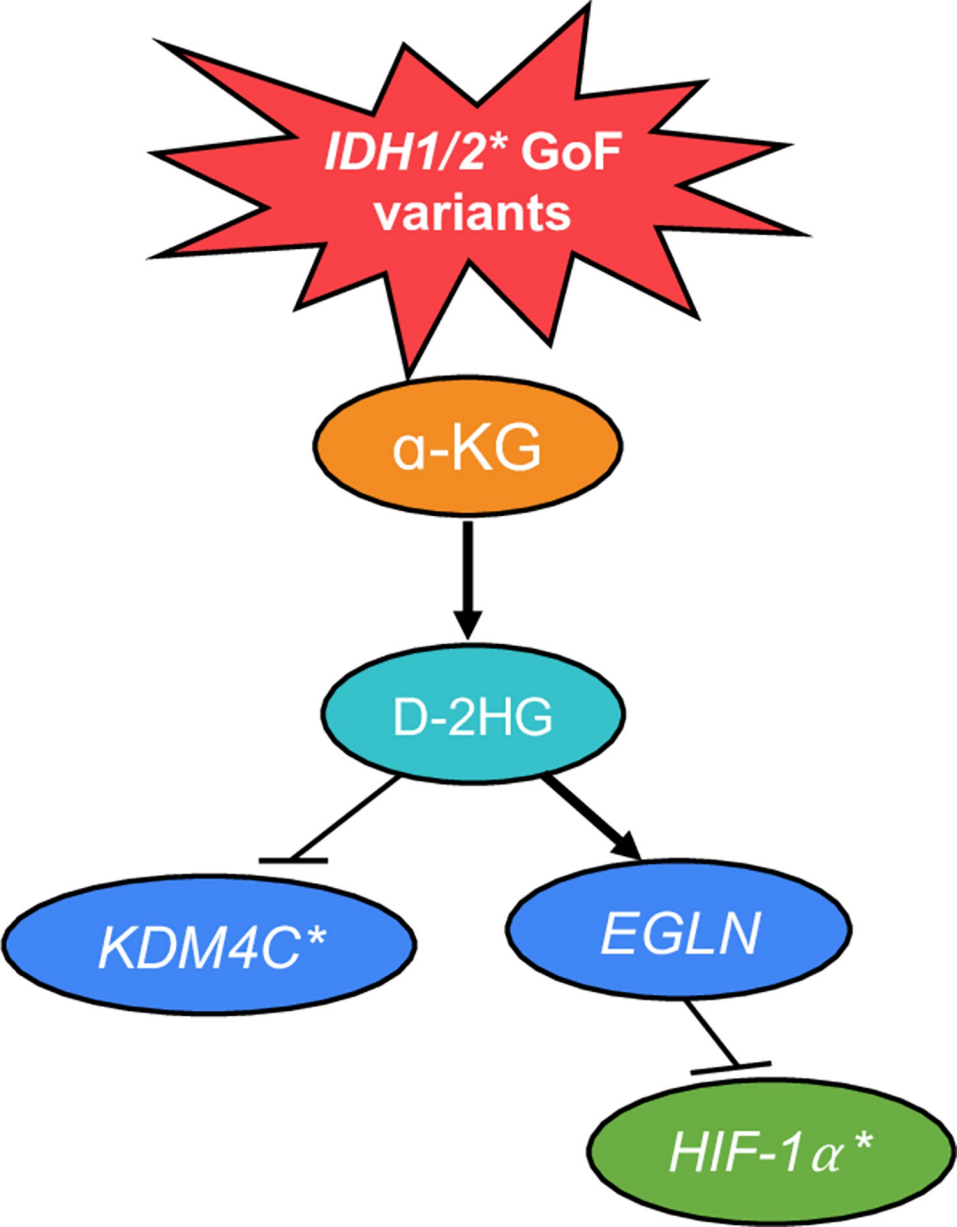
Effect of GoF *IDH1* and *IDH2* variants on *KDM4C* and HIF-1α. *Asterisks denote genes found mutated in probands with OD or MS in this study.

These same *IDH1* and *IDH2* variants have been reported in many cancers such as acute myeloid leukemia (AML) and glioma where co-occurrence with variants in other genes is a common mechanism [6–8]. In these cancers, *IDH1* and *IDH2* variants are thought to alter the catalytic function of the mutant enzymes, leading to the production of the oncometabolite D-2-hydroxyglutarate (D-2HG) from α-ketoglutarate (α-KG)[9]. D-2HG competitively inhibits α-KG-dependent enzymes, including the collagen prolyl 4-hydroxylases; the methyl cytosine hydroxylases TET1 and TET2; hypoxia-inducible factor (HIF) asparaginyl hydroxylase FIH1 (HIF1AN); and the Jumonji-C domain histone lysine demethylases (e.g. JMJD2C, encoded by *KDM4C*) [9,10]. Moreover, Koivunen et al. (2012) suggested that, in IDH-mutant gliomas, D-2HG increases the activity of prolyl hydroxylase domain protein 2 (encoded by *EGLN1*), which subsequently decreases HIF activity [11] (Fig 1). Based on the co-occurrence of *IDH1* and *IDH2* variants with variants in other genes in AML and glioma and on the fact that ~50% of the probands with OD and MS develop a malignancy including leukemia and glioma; we investigated the germline samples from probands with OD and MS for additional causative variants.

To search for possible causative germline or early post-zygotic variants responsible for OD and/or MS, we performed whole exome sequencing (WES) or whole genome sequencing (WGS) on germline DNA from 94 probands (71 OD and 23 MS). We identified rare (minor allele frequency [MAF] <1%) candidate causative variants in 6 genes (*HIF1A*, *VHL*, *IDH1*, *IDH2*, *KDM4C*, and *CDKN2A*) related to the HIF-1 pathway in approximately 22% of the probands [21/94 total, 14/71 OD (~20%) and 7/23 MS (~30%)]. A burden analysis using the 94 probands assigned as cases and 2,054 unrelated individuals presenting no OD- or MS-related features as controls, found that variants in *HIF1A*, *VHL*, and *IDH1* were significantly enriched in cases compared to controls. To better understand the functional consequences of these variants, we investigated the downstream effects in the HIF-1 pathway by performing RNA-sequencing (RNA-seq) on fibroblast cell lines from 4 probands (2 OD and 2 MS). Hypoxia exposure altered the expression of many genes, as expected, in both proband and control cells; however, the set of differentially expressed genes in proband fibroblasts included a significantly reduced number of HIF-1 regulated genes as compared to controls. Our findings suggest that germline or early post-zygotic missense variants in *HIF1A*, *VHL*, and *IDH1* lead to susceptibility to OD and MS in approximately 22% of probands by causing HIF-1 pathway dysregulation.

## Subjects and methods

### Ethics statement

Our study was approved by the Johns Hopkins Medicine Institutional Review Board, and we obtained written informed consent from all probands and family members who participated.

### Subjects

We recruited 71 probands with OD and 23 probands with MS and their unaffected relatives through private Facebook groups and through the Johns Hopkins Hospital clinics. Our cohort was submitted to the Baylor-Hopkins Center for Mendelian Genomics (BHCMG) project for WES and for the Gabriella Miller Kids First Pediatric Research Program for WGS. Probands were eligible for inclusion in this study if they had at least 3 radiologically recognized enchondromas. All participants with MS also had one or more vascular anomalies.

### Whole Exome Sequencing (WES)

As part of the BHCMG project, we performed WES on 33 probands and their unaffected parents when available as previously described with minor alterations [12]. Briefly, DNA was isolated from whole blood or saliva of each proband and any available family members using the Puregene Blood Core Kit B (Qiagen, 158467) for whole blood and DNEasy Blood and Tissue Kit (Qiagen, 69504) for saliva. Fifty-one Mb of annotated exonic regions and flanking intronic regions were captured using the Agilent SureSelect HumanAllExonV5 Clinical Capture Reagent, then sequenced on an Illumina HiSeq2500 using HiSeq PE Cluster Kit v4. Paired-end 125 bp reads were aligned to 1000 Genomes Phase 2 (GRCh37/hg19) human genome reference build using BWA-mem 0.7.8 and GATK 3.0. Variant filtering was performed using the Variant Quality Score Recalibration method [13]. The WES mean coverage was 79X with 94.7% at 20X. The same WES method and analysis pipeline was applied to the 2,054 individuals assigned as controls for the burden analysis.

### Whole Genome Sequencing (WGS)

As part of the Gabriella Miller Kids First Pediatric Research Program, we performed WGS on 61 probands and their unaffected parents when available. DNA samples were extracted as described above. DNA samples were normalized to 1,000 ng of DNA in 50 μl of water. Following normalization, samples were acoustically sheared using a Covaris LE-220 sonicator to a final fragment size of ~350–400 bp. The sheared DNA was then transformed into a standard Illumina paired-end sequencing library via standard methods. The sheared DNA was end-repaired and A-tailed using Roche-Kapa End-Repair and A-Tailing kits under the manufacturer’s recommended conditions. Standard Illumina paired-end adaptors were ligated to the A-tailed DNA. Following ligation, the reactions were purified using AMPure XP beads. The purified ligated DNA was amplified via PCR using Roche KAPA HIFI PCR reagents using 4 cycles of PCR. The primers used in the PCR step introduced 8-base, unique, dual indexes in the i5 and i7 positions to allow sample identification/demultiplexing following sequencing. The final library was quality controlled using size verification via PerkinElmer LabChip GX and real-time PCR using the Roche KAPA SYBR FAST qPCR Master Mix, primers and standards according to the manufacturer’s directions. Libraries were normalized to 1.4 nM stocks for use in clustering and sequencing. All sequencing was performed on the Illumina NovaSeq 6000 platform by loading pooled samples to the equivalent loading of 24 samples per flow cell. Following sequencing, all base calling was performed using standard Illumina software to generate the final FASTQ files for each sample. Alignment and variant calling were performed via the Edico/Illumina DRAGEN pipeline to verify coverage and performance. Samples yielded a minimum of 440M paired-end reads at 150 nt read length with a mean coverage of 32X with 86.5% at 20X.

### Variant prioritization

#### Variant filtering

Using the PhenoDB Variant Analysis Tool [14], we prioritized rare variants (MAF <1%) from the WES and WGS data. MAFs were obtained from the Exome Variant Server (release ESP6500SI-V2), 1000 Genomes Project (The 1000 Genomes Project Consortium), gnomAD [15], and in our in-house BHCMG samples. The variants included functional (missense, nonsense, stop-loss, splice site variants and coding indels) heterozygous and homozygous variants in each proband (S1 Fig).

#### Analysis strategy

For each proband, we selected heterozygous or homozygous variants that were rare (MAF < 1%) and in coding exons (missense, nonsense, stoploss, or indels) or canonical splice sites independent of the inheritance status. From among these, we selected the variants in genes associated with phenotypes characterized by enchondromas (*PTPN11*, *ACP5*, *NKX3-2*, *IDH1*, *IDH2*, and *PTHR1*) by cross-checking ClinVar, Human Gene Mutation Database (HGMD), and OMIM diseases.

In parallel, we performed a cohort analysis. We searched among all the genes with rare functional variants in the 94 probands for the genes that were mutated in three or more probands (S1 Fig).

#### Gene-specific burden analysis

A burden analysis was performed, as previously described [16–21], for *HIF1A*, *VHL*, *IDH1*, *IDH2*, *KDM4C*, and *CDKN2A* using 94 probands (33 WES and 61 WGS) with OD or MS, who were assigned as cases, and 2,054 unrelated individuals (analyzed by WES) presenting no features related with OD or MS, who were assigned as controls. The case-control burden analysis aimed to determine if these genes were mutated (missense, nonsense, stop loss, and splice site single nucleotide variants (SNVs) with MAF <1% in gnomAD) at a higher rate among the cases as compared to the controls. A contingency 2x2 table containing the number of individuals presenting these qualified SNVs was built for each candidate gene. The p-value was determined by Fisher’s exact test (FET). We considered a FET p-value of less than 0.05 as statistically significant (S1 Fig). Principal Component Analysis was performed to assess the ethnicity of cases and controls [21].

### Cell culture and hypoxia treatment

We cultured primary fibroblasts from skin biopsies from 4 probands with enchondromatosis: 2 probands with OD (Proband 17 with the *IDH2-*p.Asp225Asn variant; Proband 18 with the *KDM4C-*p.Tyr4Cys and *KDM4C-*p. Ala774Thr variants), and 2 probands with MS (Proband 12 with the *VHL*-p.Arg210Trp variant; Proband 21 with the *CDKN2A*-p.Ala17Gly variant) in DMEM with high glucose and pyruvate (ThermoFisher, 11995065) supplemented with 10% fetal bovine serum (Gemini Bio-Products, 900–108). Three cultures were prepared 3 separate days for each proband fibroblast cell line (3 technical replicates for each proband fibroblast cell line). We also cultured primary fibroblasts from skin biopsies from 3 unrelated unaffected controls (3 biological replicates). All cells were cultured at 5% CO_2_, 95% air (~20% O_2_) until late log phase (90% confluent), when they were separated for hypoxia treatment. Non-hypoxic control cells continued growth under standard tissue culture conditions but were supplemented with fresh media. For cells exposed to hypoxia, we added fresh media and incubated them at 1% O_2_ for 24 hours prior to RNA extraction [22].

### RNA sequencing and analysis

We isolated total cellular RNA from 4 proband (3 technical replicates for each proband) and 3 control fibroblast cell lines cultured either at 20% O_2_ (designated “normoxia”) or 1% O_2_ (designated “hypoxia”) using the RN-easy Mini Kit (Qiagen, 74104). The controls were fibroblast cell lines from 3 healthy unrelated individuals without OD or MS (3 biological replicates). The controls included male and female samples in the same age range as the probands (from 23 to 48 years of age). Libraries were prepared using the NEBNext Ultra II Library Prep Kit and sequenced on an Illumina NovaSeq 6000. The RNA sequencing from probands and controls was performed once to avoid batch effects. We performed the following data analysis steps using Partek Flow (https://www.partek.com/partek-flow/). We aligned paired-end 100-bp reads to the human genome reference build hg19/GRCh37 using STAR 2.5.3a and quantified the reads using the Partek E/M model according to RefSeq Transcripts 85 and normalized by FPKM (fragments per kilobase of exon per million reads). We added 0.0001 to all read counts to allow for statistical testing and performed differential expression analysis using Partek GSA in the default settings. We also analyzed raw count data generated in Partek Flow using the Partek E/M model by rounding all counts to the nearest integer, then using the DeSeq2 R package on Bioconductor [23]. We also curated a list of 836 genes known to be regulated by HIF-1 based on published studies of HIF-1α knockdown mammalian cell lines using RNAseq or ChIP-seq [24–27]. We then compared our differentially expressed gene sets from both algorithms to our set of HIF-1 regulated genes. Additionally, we performed a pathway analysis with g:Profiler using the default settings, which includes multiple-testing correction by the in-house algorithm g:SCS [28] (S5–S8 Tables). For this analysis we only included the genes found to be differentially expressed by both algorithms, Partek Flow and DeSeq2.

## Results

### Identification of Candidate Variants in Genes Related to the HIF-1 Pathway in 21 Probands with OD or MS

Our analysis of rare functional variants in genes associated with phenotypes characterized by enchondromas such as *PTPN11*, *ACP5*, *NKX3-2*, *IDH1*, *IDH2*, and *PTHR1* identified three probands with *IDH1* missense variants (2 variants; Probands 6, 13 and 14; Table 1) and three probands with *IDH2* missense variants (2 variants; Probands 15, 16 and 17; Table 1). Variants in *PTPN11*, *ACP5*, *NKX3-2*, and *PTHR1* were not identified among these individuals.

**Table 1:** Candidate causative variants in genes related to the HIF-1 pathway identified in our cohort of 94 probands with OD or MS. Minor allele frequency (MAF) for each variant was accessed from the gnomAD version v2.1.1 on October 19, 2021. Inheritance was determined by Sanger sequencing from the parents’ germline samples, if available. Father—variant inherited from unaffected father; Mother—variant inherited from unaffected mother. NA–not available. Het–Heterozygous. bold—not present in gnomAD.

ID	Disease	Gene mutated	Variant	Transcript	Genomic location (Build 37/hg19)	Zygosity	MAF	Inheritance	Malignancies	CADD score
1	MS	*HIF1A*	p.Val74Leu	NM_001243084:c.G220C	Chr14:62187212	Het	1.94e-3	Mother	No	23.0
2	OD	*HIF1A*	**p.Pro239Leu**	NM_001243084:c.C716T	Chr14:62194244	Het	0	Mother	No	25.2
3	OD	*HIF1A*	p.Asp446Tyr	NM_001243084:c.G1336T	Chr14:62204819	Het	3.56e-3	Mother	No	22.1
4	OD	*HIF1A*	p.Arg655His	NM_001243084:c.G1964A	Chr14:62207705	Het	4.24e-5	Father	No	14.22
5	MS	*HIF1A*	**p.Ala678Val**	NM_001243084:c.C2033T	Chr14:62207774	Het	0	Father	No	21.6
6	OD	*HIF1A*	**p.Ser716Cys**	NM_001243084: c.C2147G	Chr14: 62207888	Het	0	Father	Glioma	1.008
		*IDH1*	**p.Arg132His**	NM_005896:c.G395A	Chr2: 209113112	Mosaic (Total number of reads – 54; VAF – 37%; Saliva sample)	0	de novo		24.9
7	MS	*HIF1A*	**p.Glu481Lys**	NM_001243084: c.G1441A	Chr14: 62204924	Het	0	NA	No	25.0
		*VHL*	p.Pro25Leu	NM_000551: c.C74T	Chr3: 10183605	Het	2.99e-3			16.75
8	OD	*VHL*	p.Pro25Leu	NM_000551: c.C74T	Chr3: 10183605	Het	2.99e-3	Father	No	16.75
9	OD	*VHL*	p.Glu52Lys	NM_000551: c.G154A	Chr3: 10183685	Het	8.52e-5	NA	No	14.67
		*IDH1*	**p.Arg132His**	NM_005896: c.G395A	Chr2: 209113112	Mosaic (Total number of reads – 46; VAF – 32%; Enchondroma sample)	0			24.9
10	OD	*VHL*	p.Pro81Ser	NM_000551: c.C241T	Chr3: 10183772	Het	2.18e-4	Father	No	23.0
11	OD	*VHL*	p.Ile180Val	NM_000551: c.A538G	Chr3: 10191545	Het	1.06e-5	NA	No	24.8
12	MS	*VHL*	p.Arg210Trp	NM_000551: c.C628T	Chr3: 10191635	Het	3.59e-5	NA	Nasopharyn-geal carcinoma undifferen-tiated type	15.3
13	OD	*IDH1*	**p.Arg132His**	NM_005896: c.G395A	Chr2: 209113112	Mosaic (Total number of reads – 76; VAF – 39%; Saliva	0	de novo	Glioma	24.9
14	OD	*IDH1*	p.Ile189Val	NM_001282386: c.A565G	Chr2: 208243560	Het	3.68e-4	Mother	No	4.69
15	OD	*IDH2*	p.Thr435Met	NM_002168: c.C1304T	Chr15: 90627553	Het	3.61e-3	Father	No	16.01
16	OD	*IDH2*	p.Thr435Met	NM_002168: c.C1304T	Chr15: 90627553	Het	3.61e-3	Mother - Het Father - Het	No	16.01
17	OD	*IDH2*	p.Asp225Asn	NM_002168: c.G673A	Chr15:90631596	Het	2.87e-4	NA	No	24.6
18	OD	*KDM4C*	p.Tyr4Cys	NM_001146696: c.A11G	Chr9: 6720959	Het	1.08e-5	Father	No	3.376
			p.Ala774Thr	NM_001146695: c.G2320A	Chr9: 7049096	Het	1.77e-5	Mother		26.0
19	MS	*KDM4C*	p.Arg371Gln	NM_001146695: c.G1112A	Chr9: 6981115	Het	2.29e-3	Not in Mother, Father NA	Melanoma and chondrosarcoma	3.622
		*CDKN2A*	p.Ala121Thr	NM_058195: c.G361A	Chr9: 21971040	Het	2.25e-4	Mother, Father NA		9.677
20	MS	*CDKN2A*	p.Ala121Thr	NM_058195: c.G361A	Chr9: 21971040	Het	2.25e-4	Mother	No	9.677
21	MS	*CDKN2A*	**p.Ala17Gly**	NM_000077: c.C50G	Chr9: 21974777	Het	0	NA	No	12.08

The cohort analysis of all genes with variants in 3 or more probands uncovered an additional four genes, *HIF1A* (7 variants; Probands 1–7; Table 1), *VHL* (5 variants; Probands 7–12; Table 1), *KDM4C* (3 variants; Probands 18 and 19; Table 1), and *CDKN2A* (2 variants; Probands 19–21; Table 1) with germline, heterozygous, rare, missense variants.

Taken together, we identified six candidate genes with 21 distinct germline or early post-zygotic, rare variants (total 25 variants) in 21 probands (14 with OD and 7 with MS). All were rare (gnomAD MAF < 0.005) missense variants, including four novel *HIF1A* variants and one novel *CDKN2A* variant (Table 1). All eight genes have missense Z-scores from -0.39 to 2.22 (*HIF1A* – 2.22; *IDH1*–0.6; *IDH2*–1.34; *VHL*—-0.39; *KDM4C* –-0.49; *CDKN2A* –-1.01). However, these genes, which do tolerate some pathogenic variants, may yet be excellent candidates for OD and MS since this disorder is not a lethal or negatively selected phenotype; in other words, the missense Z-scores is not indicative of its potential phenotypic role when the trait is not lethal and could result from accumulated effects of multiple genes (i.e., an oligogenic disease) [29].

The *IDH1*-p.Arg132His variant in Probands 6 and 13 was identified with WES performed using DNA extracted from saliva and had frequencies of ~37% and ~39% among the total number of reads, respectively. The *IDH1*-p.Arg132His variant occurred *de novo* in Proband 6 and 13. Proband 14 was heterozygous for the *IDH1*-p.Ile189Val variant inherited from the mother (Table 1).

Proband 8 was heterozygous for the *VHL*-p.Pro25Leu variant and Proband 10 was heterozygous for the *VHL*-p.Pro81Ser variant, each inherited from an unaffected father. Probands 7, 9, 11, and 12 were heterozygous for the *VHL*-p.Pro25Leu, *VHL*-p.Glu52Lys, *VHL*-p.Ile180Val, and *VHL*-p.Arg210Trp variants, respectively, but parental samples were not available for segregation analysis (Table 1).

Probands 1, 2, 3, 4, 5, and 6 were heterozygous for the *HIF1A*-p.Val74Leu, *HIF1A*-p.Pro239Leu, *HIF1A*-p.Asp446Tyr, *HIF1A*-p.Arg655His, *HIF1A*-p.Ala678Val, and *HIF1A*-p.Ser716Cys variants, respectively, each inherited from an unaffected parent. Proband 7 was heterozygous for the *HIF1A*-p.Glu481Lys variant, but parental samples were not available for segregation analysis (Table 1).

Probands 15 and 16 were heterozygous for the *IDH2*-p.Thr435Met variant. Proband 15 inherited this variant from his unaffected father. In the family of Proband 16, both parents were heterozygotes for the *IDH2*-p.Thr435Met variant. Proband 17 was heterozygous for the *IDH2*-p.Asp225Asn variant, and parental samples were not available (Table 1).

Proband 18 was compound heterozygous for the *KDM4C*-p.Tyr4Cys (inherited from the father) and *KDM4C*-p.Ala774Thr (inherited from the mother) variants. Proband 19 was heterozygous for the *KDM4C*-p.Arg371Gln variant. This variant was not present in his mother; DNA from the father was not available.

Proband 19 was also heterozygous for the *CDKN2A*-p.Ala121Thr variant, which was carried by the unaffected mother. DNA from the father was not available. Proband 20 was also heterozygous for the *CDKN2A*-p.Ala121Thr variant, which was inherited from his mother. Proband 21 was heterozygous for the *CDKN2A*-p.Ala17Gly variant; parental DNA was not available.

In three probands (6, 7, and 19; Table 1), we identified candidate germline or early post-zygotic variants at two loci. Proband 6 was heterozygous for a germline variant, *HIF1A*-p.Ser716Cys (inherited from the father), and heterozygous for a *de novo IDH1*-p.Arg132His variant, which was identified in saliva by WES with a variant frequency of ~37%. Proband 7 was heterozygous for the *HIF1A*-p.Glu481Lys and *VHL*-p.Pro25Leu variants; parental DNA was not available. Proband 19 was heterozygous for the *KDM4C*-p.Arg371Gln and *CDKN2A*-p.Ala121Thr variants; his mother was heterozygous for the *CDKN2A* variant but not the *KDM4C* variant, while paternal DNA was not available.

Proband 9 had the germline heterozygous *VHL*-p.Glu52Lys variant and a somatic *IDH1*-p.Arg132His variant, which was identified in DNA from an enchondroma but not in DNA from blood. Enchondroma samples from two other probands with HIF-1 pathway variants (Probands 7 and 12, Table 1) were available for sequencing. We investigated *IDH1* and *IDH2* variants in these samples by Sanger and/or WES and did not identify any rare coding *IDH1* or *IDH2* variants.

Sanger sequencing was performed to validate the presence of the candidate variants and for family segregation. DNA was not available for Sanger sequencing of the variants found in Probands 15, 20 and 21. All the other candidate variants were confirmed by Sanger sequencing. Primer pairs designed for genomic DNA amplification and sequencing are reported in the Supplementary Material (S9 Table).

### Gene-specific burden analysis

The burden analysis identified a statistically significant difference when comparing the proband group to the control group for three of the six genes tested. *HIF1A* was mutated in seven out of 94 probands (7.4%) and in 37 individuals in the control group (1.8%). The variants identified among the probands were not found among the controls and five out of seven variants had CADD >21 and were considered potentially damaging [30]. Using a 2x2 contingency table, we found a p-value of 0.002 with an OR = 4.3 and a 95% confidence interval ranging from 1.9 to 10.1 (Table 2). *VHL* was mutated in six out of 94 probands (6.4%) and in eight individuals in the control group (0.4%). The variants identified among the probands were not found among the controls, and three of the five distinct variants have been previously described as causative of Von-Hippel-Lindau (VHL) syndrome (*VHL*-p.Glu52Lys; *VHL*-p.Pro81Ser; and *VHL*-p.Ile180Val) [31–33]. The *VHL*-p.Arg210Trp variant is classified as pathogenic at the UMD-VHL mutations database (http://www.umd.be/VHL/) and, while it has not been described as causative of VHL syndrome, it has been described as causative of oligoastrocytoma at the VHLdb (http://vhldb.bio.unipd.it/mutations). Using a 2x2 contingency table, we found a p-value of 1.36e-05 with an OR of 17.4 and a 95% confidence interval ranging from 5.9 to 51.3 (Table 2). *IDH1* was mutated in three out of 94 probands (3.2%) and in 11 individuals in the control group (0.5%). The variants identified among the probands were not found among the controls, and two probands had the known GoF *IDH1*-p.Arg132His variant. Using a 2x2 contingency table, we found a p-value of 0.02 with an OR of 6.1 and a 95% confidence interval ranging from 1.6 to 22.3 (Table 2). Principal Component Analysis supports that these differences were not due to different ethnic backgrounds of cases and controls (S2 Fig).

**Table 2:** Burden analysis results showing statistically significant difference when comparing the number of rare functional variants in the proband group to the control group for *HIF1A*, *VHL*, and *IDH1*.

Group	Proband samples	Control samples	p (FET)	OR (95% CI)
With *HIF1A* variants	7	37	0.002	4.3 (1.9 to 10.1)
Without *HIF1A* variants	87	2017		
With *VHL* variants	6	8	1.36e-05	17.4 (5.9 to 51.3)
Without *VHL* variants	88	2046		
With *IDH1* variants	3	11	0.02	6.1 (1.6 to 22.3)
Without *IDH1* variants	91	2043		

### RNA-seq analysis of proband fibroblast RNA reveals fewer HIF-1 regulated genes as differentially expressed in response to hypoxia

**Fig 2:**
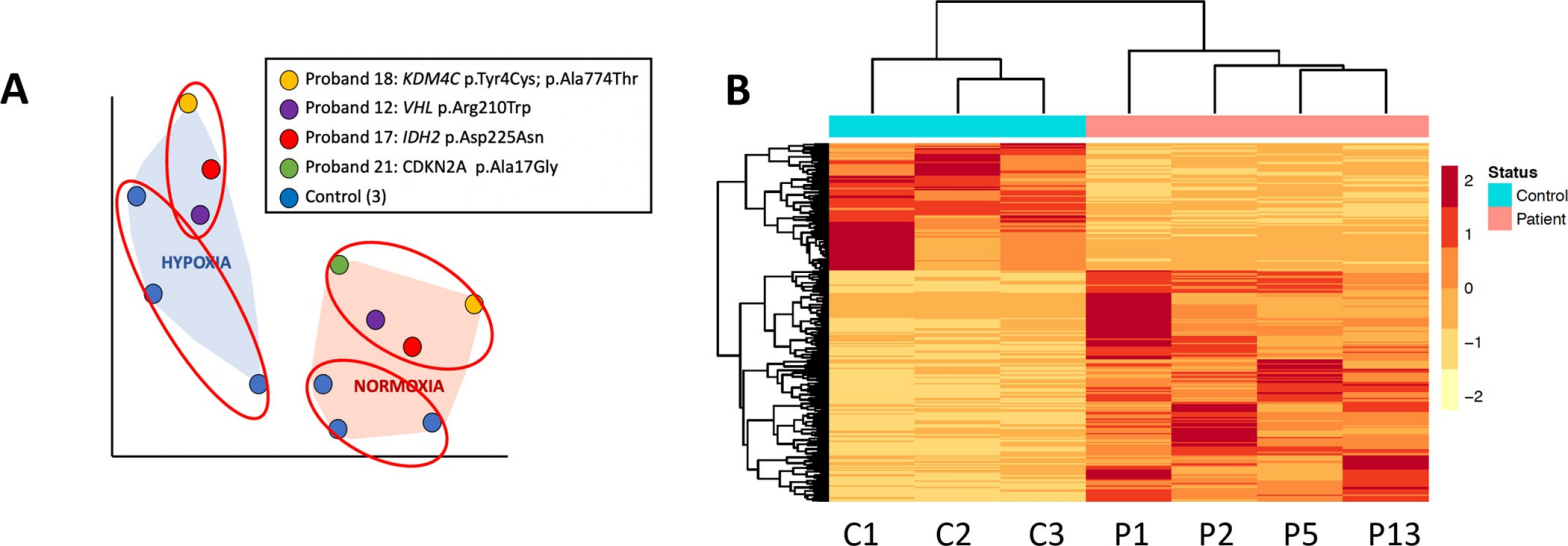
RNA-seq analysis shows differentially expressed genes between proband and control groups. A. T-SNE shows clusters based on hypoxia treatment and whether the sample is from a proband or a control. B. Heatmap of significant (p<0.05) differentially expressed genes in 4 probands at normoxia vs. 3 controls at normoxia. Proband numbers (P1-P4) correspond to Table 1.

We analyzed the transcriptomes of proband (Probands 12, 17, 18, and 21; Table 1) and control fibroblasts using T-stochastic neighbor embedding (T-SNE). We found that the most significant differences were between the fibroblasts cultured at 20% O_2_ (“normoxia”) and those exposed to 1% O_2_ (“hypoxia”) for 24 hours (Fig 2A and 2B). Additionally, the results from proband fibroblasts clustered together within their respective culture groups, as did control cells (Fig 2A and 2B). Differential expression analysis by DESeq2 and Partek Flow identified genes with significantly different fold changes between groups (i.e., proband vs. control, hypoxia vs. normoxia). Genes were considered differentially expressed if expression changes reached a significance level of p < 0.05 after multiple testing correction using both algorithms (Tables 3 and S1–S4).

**Table 3:** The numbers of differentially expressed genes with a p-value less than 0.05 are shown for each comparison. See S1–S4 Tables for full lists of significant differentially expressed genes.

Test Sample (#)	vs.	Control Sample (#)	Partek Genes p<0.05 (Total: # up, # down)	DESeq2 Genes p<0.05 (Total: # up, # down)	Both Methods Genes p<0.05 (Total: # up, # down)
OD/MS, normoxia (4)	vs.	Control, normoxia (3)	470: 75 ↑, 395 ↓	273: 176 ↑, 97 ↓	58: 25 ↑, 33 ↓
OD/MS, hypoxia (3)	vs.	Control, hypoxia (3)	640: 253 ↑, 387 ↓	196: 80 ↑, 116 ↓	28: 10 ↑, 18 ↓
OD/MS, hypoxia (3)	vs.	OD/MS, normoxia (3)	1319: 239 ↑, 1080 ↓	1172: 642 ↑, 530 ↓	363: 195 ↑, 168 ↓
Control, hypoxia (3)	vs.	Control, normoxia (3)	1492: 160 ↑, 1332 ↓	441: 302 ↑, 139 ↓	154: 95 ↑, 59 ↓

**Fig 3:**
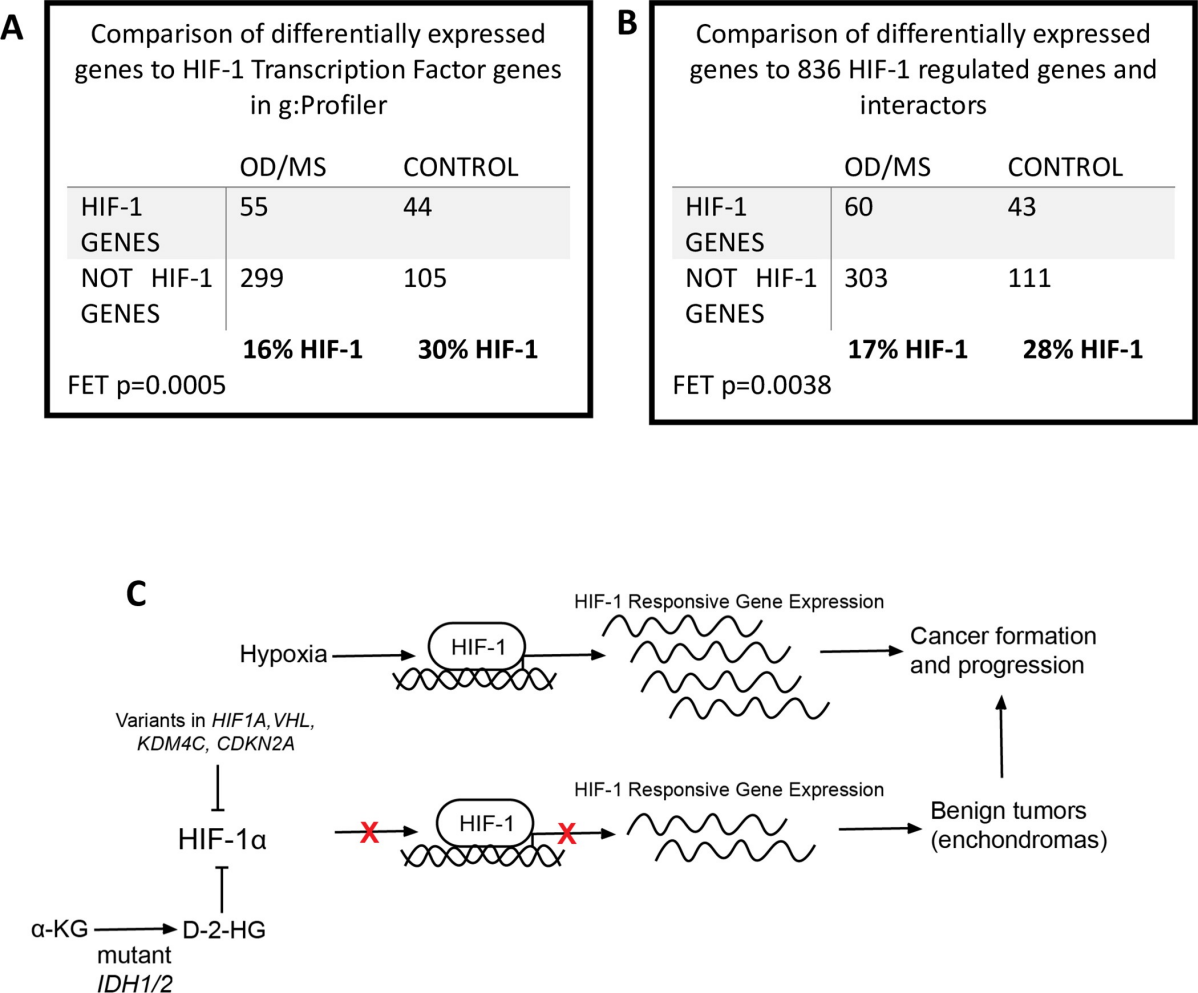
A. RNA-seq comparison of hypoxia-induced differentially expressed genes from primary fibroblasts from probands at hypoxia and hypoxia-induced differentially expressed genes from control fibroblasts at hypoxia (Table 3) to genes annotated as being regulated by the HIF-1 transcription factor by g:Profiler. B. RNA-seq comparison of hypoxia-induced differentially expressed genes from primary fibroblasts from probands at hypoxia and hypoxia-induced differentially expressed genes from control fibroblasts at hypoxia (Table 3) to curated list of HIF-1 regulated genes and interactors. C. Proposed mechanism of the role of the candidate causative variants in the HIF-1 pathway and the formation of enchondromas, vascular anomalies and chondrosarcomas in probands with OD and MS.

To interrogate the hypoxia response in proband and control fibroblasts, we compared gene expression of these cells cultured at normoxia to passage-matched cultures of the same lines after 24 hours at hypoxia. As expected, hypoxia exposure altered the expression of many genes in both proband and control fibroblasts. In control cells, 29.5% of the genes differentially expressed in response to hypoxia (44/149) were regulated by HIF-1 as determined by g:Profiler (Fig 3A). HIF-1 was the #3 and #4 annotated transcription factor match by g:Profiler (S8 Table). Interestingly, in proband cells, HIF-1 regulated only 15.5% of the genes (55/354) (Fig 3A), and HIF-1 was a much lower transcription factor match (#192) by g:Profiler (S7 Table). We also used our curated list of 836 HIF-1 regulated genes [24–27] in parallel with the g:Profiler analysis to confirm our findings. We found similar results in our curated list: 17.1% (60/363) of differentially expressed genes in proband cells were HIF-1 regulated, while 28% (43/154) were HIF-1 regulated in control cells (Fig 3B). These parallel analyses suggest that in proband fibroblasts, the response of the HIF-1 regulated genes was impaired, since the percentage of HIF-1 regulated genes among the genes differentially expressed in response to the hypoxia treatment was ~50% less in the proband cells.

### RNA-seq of proband fibroblasts reveals decreased expression of HIF-1 regulated genes in response to hypoxia

Next, we compared mRNA expression of HIF-1 regulated genes in hypoxic fibroblasts from probands and controls. Twenty-eight mRNAs were differentially expressed. Of these 28 mRNAs, 7 are products of known HIF-1 regulated genes (*HES1*, *HSPA2*, *IPO13*, *LBH*, *NR2F2*, *SMAD7*, *TGM2*) with all 7 (100%) showing decreased expression in proband cells, consistent with decreased HIF-1 activity (S2 Table).

Finally, we identified 15 known HIF-1 regulated genes with > 3-fold increased expression in response to hypoxia in control cells (Partek analysis). All 15 had decreased expression in hypoxic proband fibroblasts (*ALDOC*, *ANGPTL4*, *APLN*, *BNIP3*, *CA12*, *ENO2*, *GYS1*, *INHBB*, *miR210HG*, *NDRG1*, *PPP1R3G*, *STC1*, *SYNPO*, *VEGFA*, *ZNF395*) (S3 and S4 Tables).

## Discussion

Here, we present data supporting involvement of the HIF-1 pathway in the development of two rare cancer susceptibility disorders, OD and MS. We found 25 rare germline or early post-zygotic missense variants in *HIF1A*, *VHL*, *IDH1*, *IDH2*, *KDM4C*, and *CDKN2A* in 21 probands with OD or MS. The burden analysis showed that rare SNVs in *HIF1A*, *VHL* and *IDH1* were more common in the probands than in the controls with a statistically significant difference; furthermore, none of the specific SNVs identified in probands were identified in controls. We also identified the known GoF *IDH1*-p.Arg132His variant in saliva samples from 2 probands using WES. Additionally, in 4 of the 21 probands, we identified candidate susceptibility variants at two different loci encoding HIF-1 pathway components.

Although the GoF variants in *IDH1* and *IDH2* have been previously identified in the tumors of ~80% of individuals with OD and MS [4, 5], their role in the tumor formation in these individuals is unclear. *IDH1* and *IDH2* participate in many cellular processes, especially through production of α-KG, which is a key substrate for a large family of dioxygenases. By using α-KG as a substrate to produce D-2HG, the known GoF variants in *IDH1* or *IDH2* dysregulate the activity of multiple dioxygenases, including several in the HIF-1 pathway [11,34–37] (Fig 1).

OD and MS are characterized by abnormal cartilage and vascular development and tumors. Mice with homozygous conditional knockout of *Hif-1α* in osteoblasts demonstrate markedly decreased trabecular bone volume, reduced bone formation rate, and abnormal cortical bone architecture [38], whereas conditional knock-out of *Hif-1α* in chondrocytes causes cell death in the cartilaginous growth plate of developing long bones [39]. In tumors, HIFs stimulate blood vessel formation and reprogram metabolism for effective proliferation of cancer cells [40]. Recent studies indicate that increased HIF-2α activity is associated with malignant progression in chondrosarcoma [41]. It is possible that these tumors may behave similar to renal cell carcinoma (RCC) in which HIF-2α GoF and HIF-1α loss-of-function (LoF) occur during cancer progression [42]. However, germline variants at the *HIF1A* locus have not been linked to a human disease phenotype. Here we describe seven *HIF1A* rare missense variants (including five variants not described in gnomAD) in four probands with OD and three probands with MS (Table 1). The burden analysis showed that rare functional variants in *HIF1A* were more common among the probands than among the 2,054 controls supporting the hypothesis that missense variants in *HIF1A* play a role in the pathogenesis of OD and MS. In five probands for whom the parents’ DNA samples were available, we found that the variants were inherited from one of the unaffected parents, suggesting that *HIF1A* is a susceptibility gene and that inheritance of a variant at that locus alone is not sufficient to cause OD or MS.

**Fig 4:**
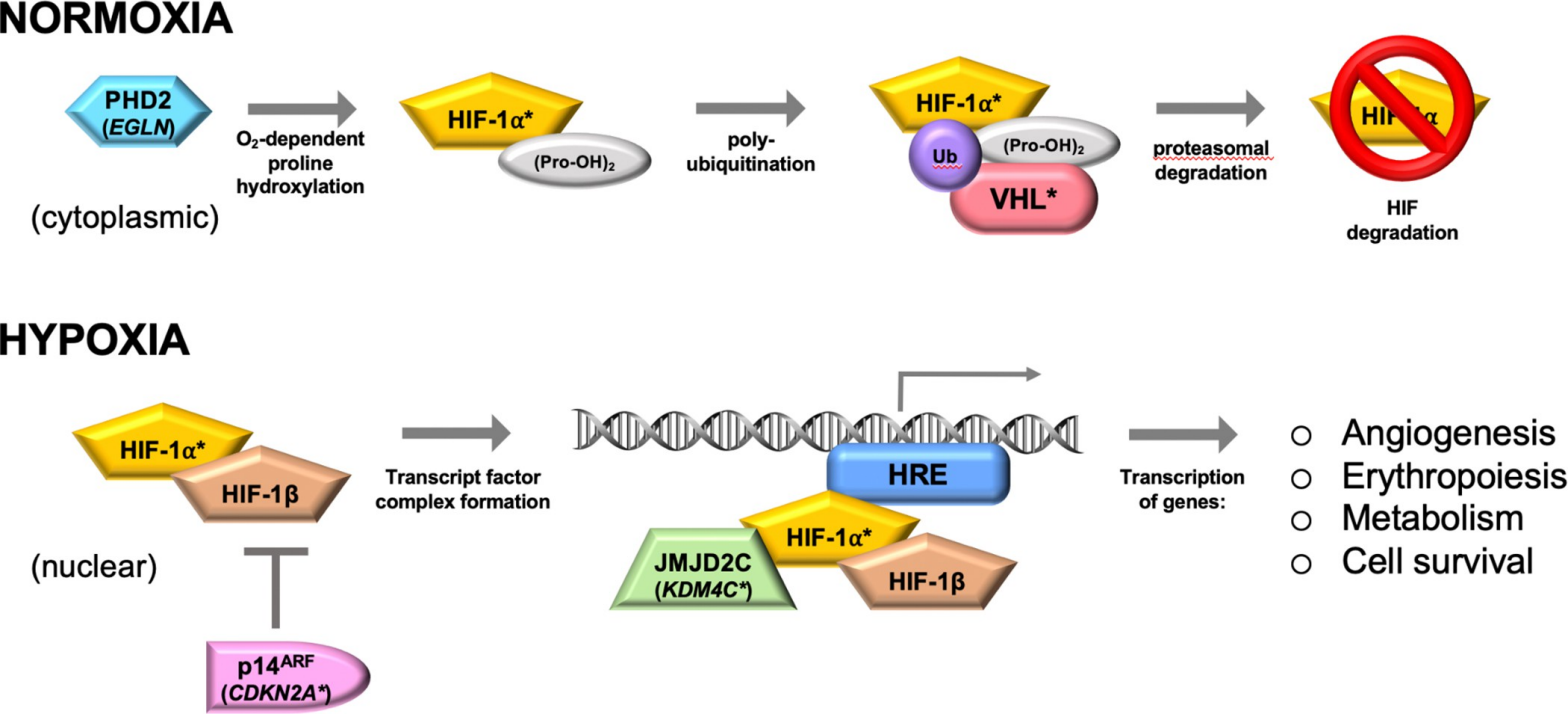
Regulation of HIF-1α degradation at normoxia and hypoxia. Asterisks denote genes found mutated in probands with OD or MS in this study.

We also identified four probands with OD and two with MS with heterozygous rare *VHL* missense variants. VHL mediates O_2_-dependent HIF-1α and HIF-2α ubiquitination, leading to proteasomal degradation under normoxia; this pathway is inhibited when O_2_ levels are low (Fig 4). Heterozygous germline LoF variants in *VHL* are associated with rare Mendelian diseases such as von Hippel-Lindau syndrome (OMIM 193300), which is a cancer susceptibility syndrome characterized by vascular anomalies such as retinal, pulmonary, liver and adrenal hemangiomas as well as the clear cell type of RCC. However, none of the known diseases caused by pathogenic variants in *VHL* are characterized by enchondromas or chondrosarcomas. Three out of the five *VHL* variants (Probands 9, 10 and 11; Table 1) have been described in association with von Hippel-Lindau syndrome and were classified as likely pathogenic or pathogenic, and the *VHL*-p.Arg210Trp variant (Proband 12; Table 1) was described in association with oligoastrocytoma and classified as likely pathogenic. However, none of our probands had features that suggest the diagnosis of von Hippel-Lindau syndrome and Proband 12 has not had a brain tumor identified by brain MRI. In 2 probands for whom the parents’ DNA sample were available (Probands 8 and 9) we found that the variants were inherited from one of the unaffected parents. These findings suggest that inheritance of a variant at the *VHL* locus alone is not sufficient to cause OD or MS. The burden analysis showed that rare functional variants in *VHL* were more common among the probands than among the 2,054 controls, supporting the hypothesis that pathogenic variants in *VHL* play a role in the pathogenesis of OD and MS.

To date, GoF *IDH1* variants in individuals with OD or MS have only been described in tumor tissue. Here we identified the *IDH1*-p.Arg132His variant in saliva samples from Probands 6 and 13 (Table 1) with variant frequencies of ~37% and ~39%, respectively, suggesting an early post-zygotic mutational mechanism with mosaicism in these cases. An additional heterozygous, rare, germline variant, *IDH1*-p.Ile189Val (predicted to be pathogenic by DANN, EIGEN, FATHMM-MKL, LIST-S2, M-CAP, MutationAssessor, MutationTaster, and SIFT) was identified in Proband 14 (inherited from the mother); further functional studies are necessary to determine if this variant causes the same GoF as the *IDH1*-p.Arg132 variants (i.e., production of D-2HG).

In seven probands, we also found rare missense variants in *IDH2*, *KDM4C*, or *CDKN2A*. *KDM4C* is a HIF-1 coactivator and α-KG-dependent lysine demethylase that modifies histones (Fig 4). *KDM4C* knockdown in breast cancer cells has been shown to reduce expression of HIF-1 regulated genes and limit breast tumor growth and metastasis in mice [22]. *CDKN2A* encodes the protein p14^ARF^, a tumor suppressor that was reported to sequester HIF-1α in the nucleolus and thereby inhibit HIF-1-mediated transcription [43] (Fig 4). The burden analyses for *IDH2*, *KDM4C*, and *CDKN2A* did not show that rare functional variants in these genes were more common among the probands than among the controls. However, we showed that in fibroblasts from probands with germline variants in *VHL* (Proband 12), *IDH2* (Proband 17), *KDM4C* (Proband 18), and *CDKN2A* (Proband 21), the expression of HIF-1 regulated genes is impaired under hypoxia, supporting the role of variants at these loci in the pathogenesis of OD and MS. Under this hypothesis, the *VHL* variants identified in our probands would be GoF variants; however, the variants identified in Probands 9, 10, and 11 have been associated with von Hippel-Lindau syndrome, indicating that they are LoF variants that lead to increased HIF-1α and HIF-2α protein levels in tumor tissue in which the wild-type *VHL* allele is inactivated by deletion, point mutation, or methylation. The presence of both *HIF1A* and *VHL* variants in proband 7 suggests that the OD and MS are associated with HIF-1α loss-of-function coupled with HIF-2α gain-of-function. However, functional studies to investigate the effect of these variants in different cell lines and in combination with variants at other loci are necessary to determine the molecular mechanism by which these variants contribute to the pathogenesis of OD and MS.

Four probands carried variants at two distinct loci encoding HIF-1 pathway components. Proband 6 was heterozygous for the germline variant *HIF1A*-p.Ser716Cys, and the *IDH1*-p.Arg132His variant with a variant frequency of ~37%, suggesting that the combination of *HIF1A* and *IDH1* variants in this proband could be responsible for the development of OD. Proband 9 was heterozygous for the germline variant *VHL*-p.Glu52Lys in combination with somatic variant *IDH1*-p.Arg132His identified in his enchondroma DNA but not found in his blood DNA. The use of more sensitive methods for the identification of mosaicism in probands with somatic variants may show that the *IDH1* variants are, indeed, present due to early post-zygotic mutations. The combination of *VHL* and *IDH1* variants in Proband 9 could be responsible for the development of OD. Finally, Proband 7 was heterozygous for germline variants *HIF1A*-p.Glu481Lys and *VHL*-p.Pro25Leu; and Proband 19 was heterozygous for germline variants *KDM4C*-p.Arg371Gln and *CDKN2A*-p.Ala121Thr. These findings suggest that OD and MS are characterized by an oligogenic mode of inheritance, which accounts for the lack of Mendelian inheritance in families with OD or MS, since it is much less likely that more than one family member (including the offspring of a proband) would inherit the same combination of variants in different genes as well as any necessary early post-zygotic or somatic variants. Furthermore, for inherited variants, this model would explain the apparent lack of penetrance within a family and why some of the causative variants might occur more commonly than predicted based on the frequency of the phenotype. Still, under the oligogenic model, some causative variants may have been classified as benign when investigated for causality of a different phenotype, making further analysis difficult. For the cases where only one germline candidate causative variant was identified in a HIF-1 pathway-related gene, we hypothesize the existence of a second germline, early post-zygotic or somatic causative variant that has yet to be discovered.

In summary, based on WES and WGS data, we conclude that OD and MS are cancer susceptibility syndromes characterized by cartilage and vascular abnormalities and mainly caused by pathogenic variants in *HIF1A*, *VHL*, *IDH1*, and, possibly, other genes related with the HIF-1 pathway such as *IDH2*, *KDM4C*, or *CDKN2A*. Based on RNA-seq data, we suggest that missense variants at these loci cause downregulation of the HIF-1 pathway, which impairs normal cartilage development (Fig 3C). We also suggest that OD and MS are characterized by an oligogenic mode of inheritance. Extensive functional studies are needed to further test this hypothesis.

## Supporting information

S1 Fig Variant prioritization workflow. (TIF)

S2 Fig **A**: Principal component analysis using The 1000 Genomes Project. **B**: Principal component analysis to determine the ethnicity of OD and MS cases and controls.
(TIF)

S1 Table 58 differentially expressed genes as determined by both Partek GSA and DESeq2 with p<0.05. Fold change by each algorithm is given.
(PDF)

S2 Table 28 differentially expressed genes as determined by both Partek GSA and DESeq2 with p<0.05. Fold change by each algorithm is given.
(PDF)

S3 Table 363 differentially expressed genes as determined by both Partek GSA and DESeq2 with p<0.05. Fold change by each algorithm is given.
(PDF)

S4 Table 154 differentially expressed genes as determined by both Partek GSA and DESeq2 with p<0.05. Fold change by each algorithm is given.
(PDF)

S5 Table g:Profiler results from the analysis of 58 genes differentially expressed in four proband fibroblast lines cultured at normoxia compared to three control fibroblast lines cultured at normoxia. Analysis was performed using the default settings and selecting the Ensembl ID with the most annotations for each gene name. The p-value cut-off was 0.05 after g:SCS significance adjustment.
(PDF)

S6 Table g:Profiler results from the analysis of 28 genes differentially expressed in three proband fibroblast lines cultured at hypoxia compared to three control fibroblast lines cultured at hypoxia. Analysis was performed using the default settings and selecting the Ensembl ID with the most annotations for each gene name. The p-value cut-off was 0.05 after g:SCS significance adjustment.
(PDF)

S7 Table Top five g:Profiler results for each category from the analysis of 363 genes differentially expressed in three proband fibroblast lines cultured at hypoxia compared to the same three proband fibroblast lines cultured at normoxia. Analysis was performed using the default settings and selecting the Ensembl ID with the most annotations for each gene name. The p-value cut-off was 0.05 after g:SCS significance adjustment. If number of significant genes in g:Profiler term is greater than 50, *n* is shown; full lists are available upon request.
(PDF)

S8 Table Top five g:Profiler results for each category from the analysis of 154 genes differentially expressed in three control fibroblast lines cultured at hypoxia compared to the same three control fibroblast lines cultured at normoxia. Analysis was performed using the default settings and selecting the Ensembl ID with the most annotations for each gene name. The p-value cut-off was 0.05 after g:SCS significance adjustment. If number of significant genes in g:Profiler term is greater than 50, *n* is shown; full lists are available upon request.
(PDF)

S9 Table Primer pairs designed for genomic DNA amplification and Sanger sequencing of candidate variants. (PDF)
