# Supplementary figures and images for: Disruption of the HIF-1 pathway in individuals with Ollier disease and Maffucci syndrome
Source: PLoS Genet. 2022 Dec 8;18(12):e1010504. doi: 10.1371/journal.pgen.1010504 (PMC9767349; doi:10.1371/journal.pgen.1010504)

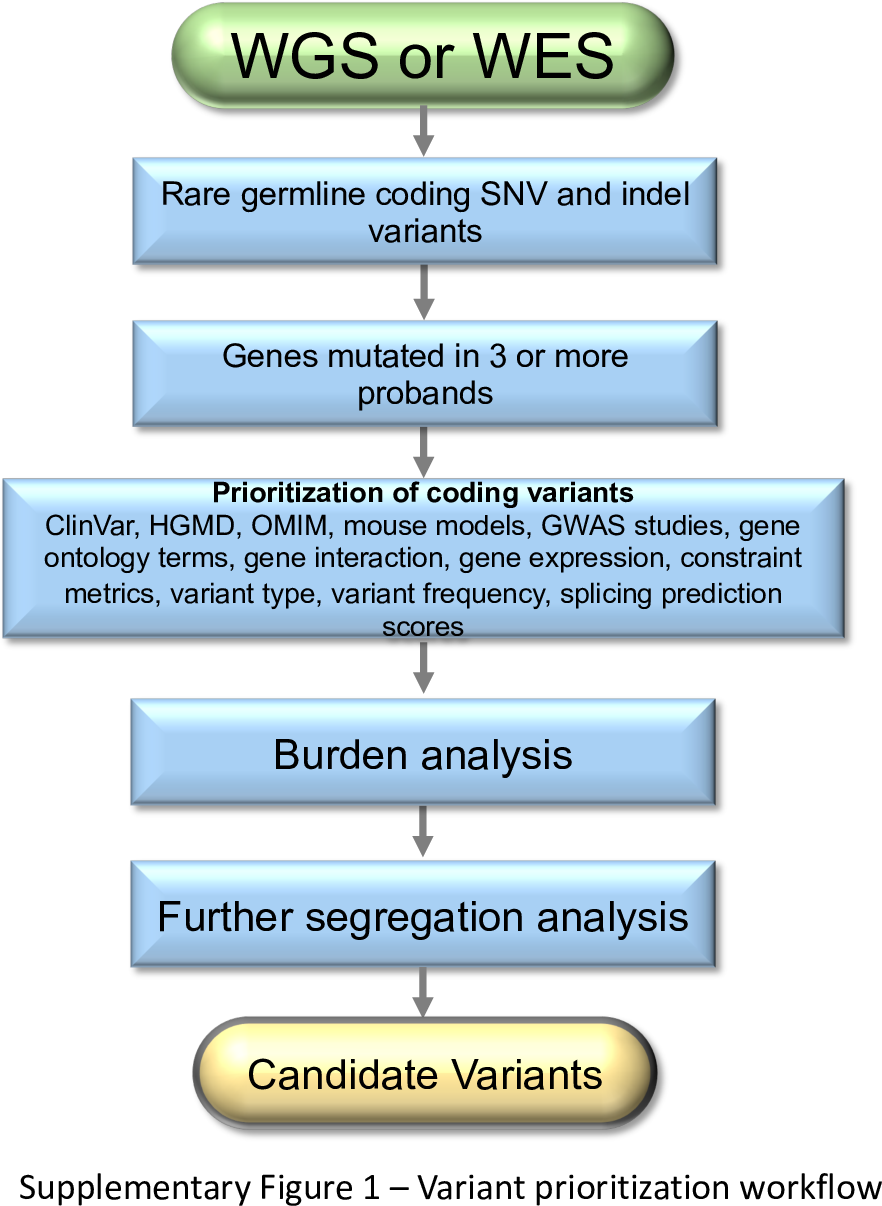

Supplement: S1 Fig — (TIF) [file pgen.1010504.s001.tif]

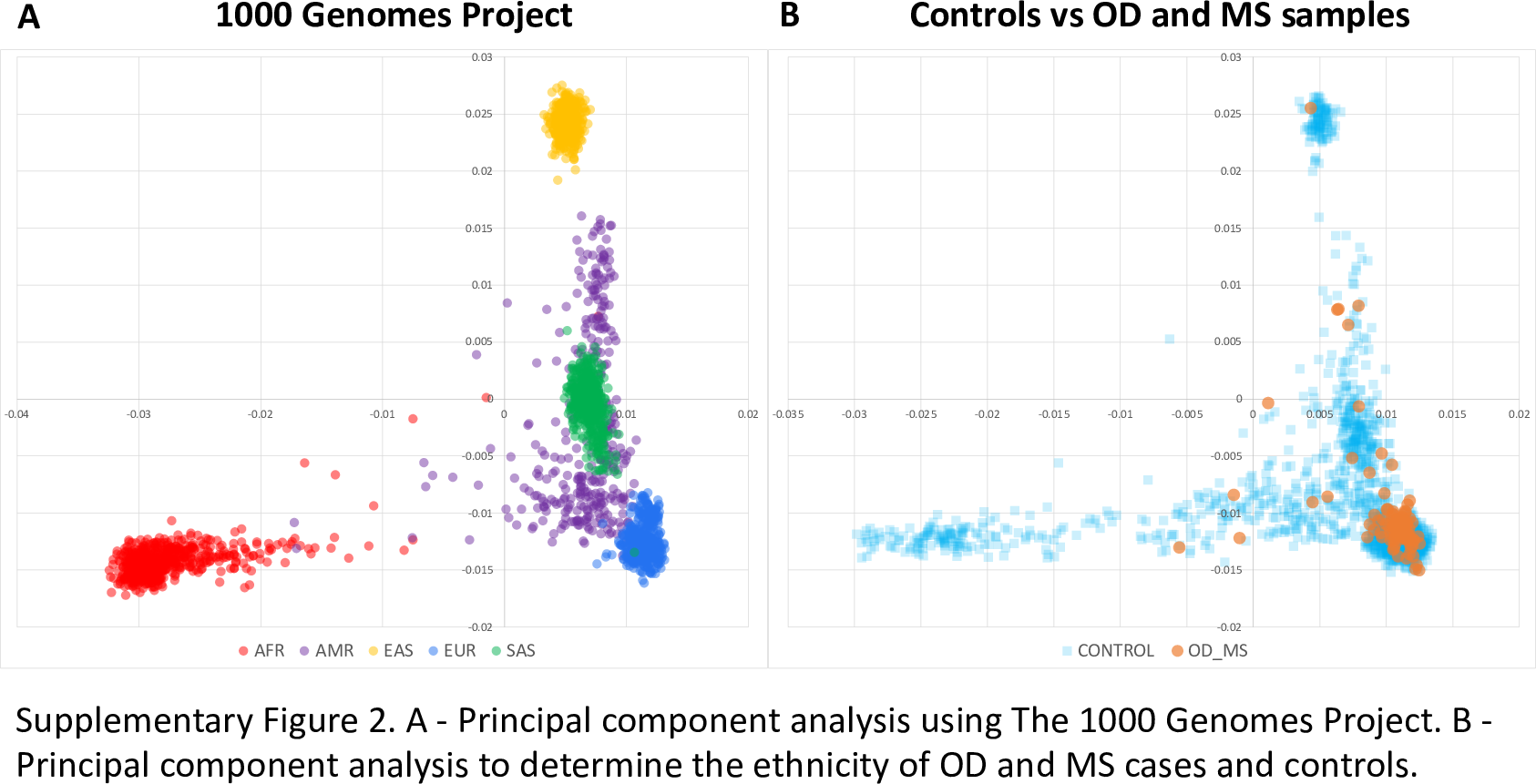

Supplement: S2 Fig — A: Principal component analysis using The 1000 Genomes Project. B: Principal component analysis to determine the ethnicity of OD and MS cases and controls. (TIF) [file pgen.1010504.s002.tif]
