# Supplementary material for: Disruption of the HIF-1 pathway in individuals with Ollier disease and Maffucci syndrome
Source: PLoS Genet. 2022 Dec 8;18(12):e1010504. doi: 10.1371/journal.pgen.1010504 (PMC9767349; doi:10.1371/journal.pgen.1010504)
Supplement: S1 Table — Fold change by each algorithm is given. (PDF) [file pgen.1010504.s003.pdf]

Genes p<0.05 by DESeq2 and Partek – Proband, Normoxia (4) vs. Control, Normoxia (3)

Gene Name	*Partek Fold Change*	*DESeq2 Fold Change*	*Gene Name*	*Partek Fold Change*	*DESeq2 Fold Change*
ACTA2	-3.97	-2.51	KLF2	-2.09	-1.86
ADAMTSL4	2.50	2.17	LMO4	1.48	1.50
AHNAK	1.72	1.74	LRP1	2.16	2.05
ANKRD1	-4.19	-2.94	MARCKSL1	2.33	2.29
BEX1	-9.21	-3.84	MRPL20	-1.55	-1.47
C1R	1.76	1.78	MYL12B	-1.45	-1.38
CALD1	-1.85	-1.70	OSMR	1.80	1.81
CAV2	-1.61	-1.52	OXTR	-4.85	-2.74
CFD	4.20	2.66	PHLDA2	-2.27	-2.01
CLTB	-1.43	-1.36	PKDCC	3.35	2.53
COL12A1	2.41	2.19	PLK3	-1.80	-1.65
CRIP1	-4.67	-2.63	PPP1R14A	-4.76	-2.82
EBNA1BP2	-1.44	-1.38	PRDX6	-1.89	-1.72
FAM43A	-3.49	-2.99	PRRC2B	1.32	1.36
FGF5	-2.22	-2.01	PRRC2C	1.36	1.40
FH	-1.545	-1.46	RHOBTB3	1.72	1.76
FLNB	1.74	1.75	RN7SL3	5.57	2.59
FTSJ1	-1.53	-1.44	RPL26L1	-1.49	-1.41
FZD1	1.84	1.81	RPS6KB2	-1.41	-1.34
GAA	2.37	2.36	RRP36	-1.39	-1.33
HES4	-8.92	-3.34	SELENOH	-1.69	-1.58
HSPB6	-2.07	-1.88	SELENOW	-1.95	-1.79
HSPG2	2.56	2.25	SEMA7A	-2.22	-1.97
IGFBP7	-4.33	-3.25	SLC25A23	2.12	2.00
IL17RA	1.46	1.48	SOX4	1.95	1.85
IL1R1	1.95	1.86	SRGN	-2.65	-2.11
ITGB1BP1	-1.43	-1.35	TIMP3	-2.38	-2.00
ITM2C	2.34	2.17	TNC	3.24	3.01
KLC1	-1.43	-1.36	WFS1	1.52	1.55

S1 Table. 58 differentially expressed genes as determined by both Partek GSA and DESeq2 with $p<0.05$. Fold change by each algorithm is given.
