# Supplementary material for: Disruption of the HIF-1 pathway in individuals with Ollier disease and Maffucci syndrome
Source: PLoS Genet. 2022 Dec 8;18(12):e1010504. doi: 10.1371/journal.pgen.1010504 (PMC9767349; doi:10.1371/journal.pgen.1010504)
Supplement: S2 Table — Fold change by each algorithm is given. (PDF) [file pgen.1010504.s004.pdf]

Genes p<0.05 by DESeq2 and Partek - Proband, Hypoxia (3) vs. Control, Hypoxia (3)

Gene Name	*Partek Fold Change*	*DESeq2 Fold Change*
BEX4	3.44	2.46
C11orf87	-9.52	-3.89
C1orf198	-3.58	-2.84
CFD	9.4	3.21
COX7A1	-2.51	-2.15
DGKQ	-1.75	-1.66
FAM43A	-2.85	-2.41
FTH1	1.81	1.80
FTH1P3	1.82	1.79
GAA	2.02	1.99
GDF6	-5.92	-3.14
GLIPR1	2.7	2.35
HES1	-9.12	-2.77
HOXA11	-7.75	-2.75
HSPA2	-3.06	-2.47
IPO13	-1.79	-1.70
ITM2C	2.18	2.02
LBH	-3.58	-2.87
LDLRAD2	2.43	2.17
MAB21L1	-6.1	-3.4
NR2F2	-3.98	-2.64
PKD2	-2.21	-2.01
PLXND1	-2.12	-1.95
PVR	2	1.92
SMAD7	-2.04	-1.90
TBX2	-7.39	-3.57
TGM2	-5.53	-3.69
TNC	2.84	2.56

S2 Table. 28 differentially expressed genes as determined by both Partek GSA and DESeq2 with p<0.05. Fold change by each algorithm is given.
