# Supplementary material for: Disruption of the HIF-1 pathway in individuals with Ollier disease and Maffucci syndrome
Source: PLoS Genet. 2022 Dec 8;18(12):e1010504. doi: 10.1371/journal.pgen.1010504 (PMC9767349; doi:10.1371/journal.pgen.1010504)
Supplement: S3 Table — Fold change by each algorithm is given. (PDF) [file pgen.1010504.s005.pdf]

Genes p<0.05 by DESeq2 and Partek - Proband, Hypoxia (3) vs. Proband, Normoxia (3)

Gene Name	*Partek Fold Change*	*DESeq2 Fold Change*	*Gene Name*	*Partek Fold Change*	*DESeq2 Fold Change*
ABCE1	-2.05	-1.79	MZT2A	1.51	1.74
ABCF2	-1.85	-1.60	NAA50	-1.59	-1.43
ABHD15	-1.96	-1.72	NAA80	1.37	1.51
ABTB1	1.56	1.81	NAP1L3	-4.66	-3.02
ACADVL	1.38	1.68	NCDN	-1.99	-1.70
ACTG1	1.35	1.53	NCKIPSD	1.27	1.60
ADAMTSL4	2.18	2.10	NCL	-1.53	-1.33
ADAR	-1.72	-1.47	NDRG1	3.1	2.96
ADPRHL2	1.39	1.69	NDUFB4	1.18	1.40
AEN	-2.47	-1.99	NDUFB8	1.28	1.56
AHSA1	-1.53	-1.35	NOL3	1.57	1.89
AKR7A2	1.23	1.46	NOL6	-2.09	-1.78
ALDH3A2	-3.18	-2.32	NOLC1	-1.94	-1.63
ALDOA	1.56	1.89	NOP14	-1.9	-1.66
ANGPTL4	5.43	3.09	NOP2	-2.88	-2.49
ANKRD37	2.02	2.00	NOP9	-1.65	-1.47
ANKRD52	-1.73	-1.47	NPM3	-2.22	-1.81
ANKZF1	1.65	2.09	NQO1	-3.63	-2.64
ANXA7	-1.75	-1.56	NR2F1	1.87	2.16
APBB1	1.34	1.56	NRN1	2.35	2.67
APEX1	-1.62	-1.40	NSUN2	-1.67	-1.46
APLN	5.04	3.16	NTAN1	1.45	1.62
ARHGAP1	-1.56	-1.40	NUDT16	-1.75	-1.49
ARHGEF25	1.48	1.71	NUDT22	1.37	1.65
ARRDC3	2.51	2.72	OAF	-1.97	-1.68
ATMIN	-1.6	-1.38	OCEL1	1.54	1.66
ATP13A1	-1.94	-1.71	ODC1	-2.53	-2.08
ATP1A1	-1.54	-1.42	OGFOD1	-1.73	-1.52
AXL	1.33	1.58	ORAI3	1.44	1.67
B3GALT4	2.94	3.31	OST4	1.39	1.55
BCKDK	2.01	2.49	OTUD1	1.62	1.93
BNIP3	2.58	3.00	P4HA2	1.87	2.06
BNIP3L	2.25	2.79	PAIP2	1.55	1.82
BOLA1	1.43	1.65	PAQR7	1.44	1.74
BRK1	1.24	1.42	PARP1	-1.71	-1.46

BTG1	2.07	2.15	PCAT6	2.15	2.97
C11orf68	1.29	1.46	PCNX3	-1.72	-1.51
C12orf49	-1.65	-1.50	PCYT2	-2.62	-2.23
C1QBP	-1.78	-1.53	PFKL	1.61	2.00
C4orf3	2.13	2.57	PFKP	1.88	2.13
CAD	-2.65	-2.28	PGAM1	1.62	1.96
CALCOCO1	1.55	1.81	PGAM4	1.65	2.03
CALHM2	1.36	1.54	PGAM5	-2.17	-1.79
CARHSP1	1.46	1.84	PGD	-2.55	-2.02
CBX6	-2	-1.70	PGK1	2.42	2.89
CCDC97	-1.85	-1.59	PI4K2A	-1.5	-1.34
CCNDBP1	1.52	1.63	PIGW	-2.57	-2.08
CCT6A	-1.85	-1.65	PIH1D1	1.3	1.53
CD276	-1.87	-1.74	PLAUR	1.6	1.63
CENPT	1.35	1.51	PLEKHB2	-1.6	-1.49
CERCAM	1.54	1.81	PLSCR3	1.35	1.58
CFLAR	1.33	1.60	PMM1	1.45	1.62
CHP1	-1.61	-1.39	PNPLA2	1.25	1.55
CHST3	-1.94	-1.73	PNRC1	1.48	1.72
CKB	3.07	2.66	POLD4	1.59	1.94
CLCN7	-2.22	-2.06	POLR2H	1.39	1.63
CLUH	-1.98	-1.76	POLR3H	-1.69	-1.49
CNDP2	-1.82	-1.55	POLRMT	-1.68	-1.45
CNOT8	1.7	2.04	PPAN	1.3	1.62
CS	-1.46	-1.31	PPM1F	-1.62	-1.41
CSNK1E	1.42	1.69	PPP1R13L	2.12	2.09
CSTF2T	-1.66	-1.43	PPP1R16A	1.64	2.02
DCAF11	-1.51	-1.34	PPP1R35	1.68	1.94
DDIT4	2.42	2.78	PPP1R3D	-1.83	-1.50
DDT	1.48	1.76	PPP1R3G	2.26	2.80
DDX1	-1.47	-1.31	PPRC1	-2.29	-1.98
DDX19A	-1.51	-1.31	PRMT5	-1.62	-1.43
DDX21	-1.98	-1.67	PRPF19	-1.85	-1.60
DDX41	2.13	2.49	PSMD11	-1.78	-1.57
DHX9	-1.65	-1.45	PSME3	-1.8	-1.56
DNAJA1	-1.78	-1.57	PSMG1	-2.05	-1.72
DOLK	-1.81	-1.57	PTPN23	-1.58	-1.40
DPYSL3	-1.66	-1.43	PTPRF	1.88	2.15
DVL2	-1.57	-1.38	PXDC1	1.48	1.72
EGLN1	1.5	1.84	PYCR3	-1.99	-1.76

EHD2	1.38	1.60	PYGB	-2.18	-1.85
EMC9	1.85	1.97	RAB29	-1.65	-1.52
ENO1	1.41	1.69	RAB32	1.31	1.52
ENO2	2.93	3.53	RANBP6	-1.99	-1.77
EPAS1	-2.71	-2.16	RASSF1	1.62	1.72
EPHX1	-2.72	-2.25	RFK	-1.78	-1.57
ERO1A	2.32	2.40	RFX5	-1.85	-1.65
ERRFI1	2.92	2.24	RGL2	1.24	1.48
ETF1	-1.55	-1.37	RGMB	1.97	2.05
EXTL3	-1.78	-1.50	RIOK3	1.57	1.81
F3	5.53	5.48	RNASE4	2.36	2.56
FAM114A1	1.28	1.44	RNASEH2C	1.51	1.66
FAM98A	-1.82	-1.65	RNF181	1.27	1.53
FASN	-3.22	-2.77	RNF208	2.29	2.79
FBXO30	-1.84	-1.60	RNF227	2.39	2.70
FCHSD1	1.44	1.61	RPS19	1.24	1.51
FEZ2	1.46	1.58	RPS26P11	1.5	1.83
FHOD1	-1.96	-1.74	RRAGA	1.34	1.55
FKBP8	1.31	1.56	RRAS	1.38	1.69
FLAD1	-1.78	-1.50	RRP36	-1.85	-1.59
FRMD8	-1.44	-1.33	RRP9	-2.44	-2.11
FUT11	1.48	1.70	RRS1	-3.51	-2.91
FYTTD1	-1.61	-1.40	RSAD1	-2.05	-1.79
G6PD	-1.75	-1.49	RTL8C	1.28	1.47
GADD45B	1.76	2.01	SAR1B	-1.66	-1.44
GAPDH	2.02	2.44	SCAMP3	-1.82	-1.62
GCN1	-1.73	-1.50	SCO2	-1.86	-1.59
GLTP	1.73	1.97	SEC61G	1.69	2.00
GNB2	1.24	1.42	SEMA7A	2.03	2.33
GNPDA1	-2.9	-2.61	SERPINE1	3.17	3.24
GORASP1	-1.6	-1.40	SIGMAR1	-1.7	-1.50
GOT2	-1.8	-1.60	SLC16A3	2.08	2.41
GPANK1	1.2	1.40	SLC27A1	1.57	1.69
GPATCH4	-2.19	-1.79	SLC27A4	-2.25	-1.93
GPI	1.49	1.92	SLC2A1	2.18	2.69
GPR176	1.23	1.56	SLC30A1	-2.23	-1.85
GSPT1	-1.49	-1.36	SLC35F6	-1.77	-1.55
GYS1	2.56	2.86	SLC38A7	-1.85	-1.68
HACD3	-1.47	-1.34	SLC48A1	-2.1	-1.63
HAGH	1.37	1.52	SMIM3	2.42	2.51

HAS2	3.85	3.68	SMYD5	-1.66	-1.42
HCFC1R1	1.93	1.97	SNHG3	-2.13	-1.88
HDAC3	1.36	1.60	SNX19	-1.75	-1.47
HILPDA	4.13	4.94	SNX21	1.51	1.73
HMBS	-2.11	-1.70	SNX33	1.64	1.90
HSD3B7	1.63	1.79	SPR	-2.06	-1.79
HSP90AB1	-1.57	-1.38	SPSB2	-3.57	-2.88
HSPA4	-2.38	-2.14	SPSB3	1.36	1.61
HSPA8	-1.64	-1.47	SQSTM1	-2.74	-2.66
HSPD1	-1.7	-1.50	SRPRA	-1.61	-1.40
HSPE1	-1.73	-1.56	SRPRB	-2.24	-1.92
HSPH1	-2.73	-2.48	SRSF9	1.18	1.43
HYAL2	-2.41	-2.04	STARD7	-1.74	-1.50
IDH1	-2.47	-1.99	STBD1	1.84	1.90
IER3	2.79	2.78	STC1	5.3	8.93
IL7R	2.57	3.52	STIP1	-1.52	-1.35
IMP4	-1.78	-1.54	STK25	1.31	1.51
INHBB	4.54	8.76	STRIP1	-1.8	-1.51
INSIG2	2.61	2.84	SYDE1	1.25	1.45
IPO13	-1.98	-1.74	SYNPO	3	2.88
IPO7	-1.7	-1.53	TALDO1	-1.63	-1.46
ITGA5	1.29	1.48	TAP2	-2.28	-1.83
JOSD2	1.41	1.62	TFRC	-3.14	-2.55
KAT2A	-1.82	-1.41	TGFBI	2.04	2.05
KCTD11	2.06	2.83	TIMP1	1.83	1.82
KCTD12	-2.32	-1.88	TIMP3	2.24	2.12
KDM6B	1.55	1.86	TKT	-2.34	-2.00
KIAA0930	-2.17	-1.83	TMEM106B	-1.71	-1.46
KIAA1191	1.55	1.84	TMEM107	1.93	2.26
KLF6	1.65	1.76	TMEM126B	-1.67	-1.53
KLHL21	-2.06	-1.90	TMEM158	3.63	2.80
KRTAP1-5	2.93	2.70	TMEM251	-2.11	-1.79
LDHA	1.78	2.13	TMSB10	2.05	2.18
LDLRAD2	2.17	2.57	TNFAIP2	1.58	1.98
LGALS1	1.49	1.77	TNIP1	1.51	1.90
LOC105369340	2.28	2.31	TOR3A	-2.37	-2.00
LOC105376805	2.28	2.17	TP53	-2.43	-1.87
LOC152048	-2.23	-2.05	TPI1	2.49	3.45
LOX	2.56	2.32	TRADD	1.56	1.77
LOXL2	1.86	2.13	TRIM26	-1.72	-1.52

LSM10	-1.92	-1.61	TRPT1	1.45	1.71
LTBR	-1.83	-1.51	TXNIP	1.68	2.06
M6PR	-1.52	-1.38	TXNRD1	-2.19	-1.81
MAFF	1.74	2.35	UBQLN1	-1.49	-1.31
MANF	1.33	1.48	UFSP1	-2.8	-2.11
MAPK7	1.49	1.75	USB1	-1.66	-1.41
MDM2	-1.6	-1.40	USP5	-1.78	-1.62
MEIS3	1.61	2.01	VARS	-2.05	-1.75
METRNL	1.55	1.84	VDAC1	1.31	1.55
METTL26	1.52	2.03	VEGFA	2.97	3.48
MIF	1.82	2.38	VEGFB	1.5	1.67
MIF-AS1	1.78	2.27	VIM	1.5	1.58
MIR210HG	2.34	3.70	VKORC1	1.69	1.87
MOSPD3	1.73	2.04	VPS18	-1.77	-1.61
MPG	1.42	1.73	VPS26B	-1.56	-1.30
MPI	1.74	2.09	VWA1	2.34	2.24
MPV17L2	-2.41	-2.03	WDR45B	1.32	1.62
MRGBP	1.32	1.68	WDR46	-1.64	-1.43
MRPL34	-1.92	-1.65	WDR54	2.12	2.75
MRPS26	-1.76	-1.50	WDR77	-1.75	-1.54
MSANTD3	1.29	1.46	WDR81	-1.87	-1.70
MT1L	3.35	2.52	WSB1	1.89	2.26
MT2A	2.34	2.08	YPEL3	1.71	2.08
MTFP1	2.03	2.27	ZFYVE19	1.24	1.44
MTMR11	1.72	1.82	ZMPSTE24	-1.5	-1.37
MYADM	1.88	2.06	ZNF395	3.03	3.59
MYBBP1A	-2.23	-1.97			

S3 Table. 363 differentially expressed genes as determined by both Partek GSA and DESeq2 with $p<0.05$. Fold change by each algorithm is given.
