# Supplementary material for: Disruption of the HIF-1 pathway in individuals with Ollier disease and Maffucci syndrome
Source: PLoS Genet. 2022 Dec 8;18(12):e1010504. doi: 10.1371/journal.pgen.1010504 (PMC9767349; doi:10.1371/journal.pgen.1010504)
Supplement: S4 Table — Fold change by each algorithm is given. (PDF) [file pgen.1010504.s006.pdf]

Genes p<0.05 by DESeq2 and Partek - Control, Hypoxia (3) vs. Control, Normoxia (3)

Gene Name	*Partek Fold Change*	*DESeq2 Fold Change*	*Gene Name*	*Partek Fold Change*	*DESeq2 Fold Change*
ADAMTSL4	2.12	2.04	MUC1	2.49	2.54
ADH5	-1.95	-1.59	MYADM	1.39	1.57
AIMP2	-2.57	-2.07	NAB2	1.39	1.57
ALDOA	1.68	1.80	NDRG1	3.53	2.86
ALDOC	4.26	2.81	NEU1	-1.99	-1.61
ALKBH5	1.55	1.69	NOL3	2	1.96
ANGPTL4	7.29	4.80	NOP2	-2.16	-1.74
ANKZF1	2.06	2.03	NOP56	-2.01	-1.65
APBB1	1.37	1.55	NPM3	-3.32	-2.42
APEX1	-1.74	-1.45	NQO1	-4.7	-2.76
APLN	19.9	4.34	OBSL1	1.4	1.59
APOL6	-2.45	-1.87	ODC1	-2.53	-1.89
ARRDC3	1.62	1.74	OSMR	1.37	1.55
BCKDK	1.76	1.97	P4HA2	1.81	1.86
BCL2L2	1.28	1.47	P4HB	1.28	1.47
BNIP3	3.81	2.96	PAQR7	1.68	1.89
BNIP3L	2.24	2.16	PCAT6	4.25	3.24
C11orf98	-2.38	-1.88	PCYT2	-2.31	-1.87
C4orf3	2.02	2.08	PFKL	1.82	1.92
CA12	4.57	3.67	PFKP	1.8	1.92
CACNB3	1.46	1.65	PGAM1	1.56	1.71
CCT6A	-1.9	-1.56	PGAM4	2	2.02
CDK4	-1.93	-1.58	PGK1	2.91	2.65
CHST2	-2.95	-2.15	PGM1	1.79	1.97
CLCN7	-1.91	-1.57	PIGY	-1.81	-1.50
CLTA	-1.8	-1.50	PLOD2	2.65	2.23
CNBP	-1.66	-1.39	PLXNA3	1.66	1.82
CNDP2	-1.69	-1.41	PPP1R13L	1.9	1.97
COX14	-1.89	-1.56	PPP1R16A	1.77	1.89
DDX21	-2.09	-1.65	PPP1R3G	3.45	3.53
DDX41	2.49	2.42	PRMT5	-1.76	-1.46
DUSP1	1.99	2.12	PRMT6	-1.91	-1.57
EGLN1	1.83	1.98	PSMC2	-1.75	-1.45
ENO1	1.57	1.75	PSMG1	-2.52	-1.94
ENO2	5.11	3.31	PTPRF	2.94	2.96
EPAS1	-2.99	-2.28	PYURF	-1.81	-1.50
ERO1A	2.46	2.51	RAB29	-2.05	-1.63

ERRFI1	3	2.25	RASSF7	2.04	2.04
FAM57A	1.57	1.74	RHBDD3	-1.96	-1.60
FTH1	-1.91	-1.60	RNASE4	2.4	2.51
FTH1P3	-1.97	-1.60	RNF208	4.12	3.39
FUT11	1.67	1.83	RRP9	-2.77	-2.15
FZD1	1.52	1.69	RRS1	-2.73	-2.10
GAPDH	2.16	2.24	RSAD1	-1.94	-1.59
GLTP	1.43	1.63	SEPHS2	-1.78	-1.48
GNPDA1	-2.64	-2.06	SERPINE1	2.34	2.44
GPATCH4	-2.72	-2.07	SLC16A3	2.69	2.62
GPI	1.76	1.82	SLC2A1	2.95	2.34
GPR176	1.33	1.53	SLC35E1	1.59	1.73
GYS1	3.03	3.03	SLC3A2	-2.09	-1.71
HAS2	2.01	2.02	SMIM3	1.67	1.87
HCFC1R1	2.02	2.00	SNHG15	-2.46	-1.88
HILPDA	3.44	2.75	SNX33	1.61	1.74
HSD17B10	-1.97	-1.62	SPSB2	-3.46	-2.24
HSPD1	-1.81	-1.50	SQSTM1	-3.18	-2.46
HSPH1	-2.14	-1.70	SRPRB	-1.92	-1.56
IGIP	1.66	1.79	SRXN1	-2.07	-1.69
ILF3-DT	-2.13	-1.70	SSSCA1	-1.83	-1.52
INHBB	10.9	6.92	STAT1	-2.25	-1.74
INSIG2	2.39	2.29	STBD1	1.86	1.92
KCTD11	2.57	2.21	STC1	9.86	4.56
KIAA1191	1.55	1.74	SYNPO	5.55	3.39
LARS	-2	-1.63	TALDO1	-2.04	-1.68
LDHA	1.72	1.82	TAP2	-2.64	-1.94
LMAN1	1.53	1.68	TBC1D9B	1.6	1.77
LOX	2.08	2.34	TOB2	1.32	1.50
LSM10	-1.85	-1.53	TPI1	2.45	2.39
MAFF	1.76	1.97	TRMT12	-2.13	-1.68
MAPK7	1.64	1.83	TXNRD1	-2.53	-1.88
MARCKSL1	1.96	2.01	VEGFA	3.95	2.95
MEIS3	1.58	1.76	VKORC1	1.59	1.73
METTL26	1.46	1.64	VWA1	3.85	2.55
MIR210HG	4.75	2.27	WDR54	1.78	1.95
MOSPD3	1.73	1.92	WSB1	2.02	2.04
MPI	1.7	1.78	XYLT2	1.3	1.49
MRPL24	-1.87	-1.54	ZNF395	4.34	3.73
MRPL52	-1.9	-1.56	ZNF593	-1.8	-1.49

S4 Table. 154 differentially expressed genes as determined by both Partek GSA and DESeq2 with $p<0.05$. Fold change by each algorithm is given.
