# Supplementary material for: Disruption of the HIF-1 pathway in individuals with Ollier disease and Maffucci syndrome
Source: PLoS Genet. 2022 Dec 8;18(12):e1010504. doi: 10.1371/journal.pgen.1010504 (PMC9767349; doi:10.1371/journal.pgen.1010504)
Supplement: S5 Table — Analysis was performed using the default settings and selecting the Ensembl ID with the most annotations for each gene name. The p-value cut-off was 0.05 after g:SCS significance adjustment. (PDF) [file pgen.1010504.s007.pdf]

Full g:Profiler Results: Proband, Normoxia (4) vs. Control, Normoxia (3)

Source	*Term*	*Adj. P*	*-log$_{10}$ Adj. P*	*Sig. Genes in Term*
GO: Molecular Function	peptide binding	0.0158	1.7986	CLTB, CRIP1, HSPG2, ITM2C, LRP1, OXTR, RPS6KB2
	structural molecule activity	0.0418	1.3786	AHNAK, CAV2, CLTB, COL12A1, HSPB6, HSPG2, IGFBP7, MRPL20, RPL26L1, TNC
GO: Biological Process	tissue development	0.0095	2.0214	ACTA2, ADAMTSL4, ANKRD1, CAV2, COL12A1, CRIP1, FLNB, FZD1, GAA, HES4, ITGB1BP1, KLF2, LMO4, PHLDA2, PKDCC, SEMA7A, SOX4, SRGN, TNC
GO: Cellular Compartment	extracellular region	0.0383	1.4163	ACTA2, ADAMTSL4, AHNAK, C1R, CFD, COL12A1, FGF5, FH, FLNB, GAA, HSPB6, HSPG2, IGFBP7, IL17RA, IL1R1, ITM2C, MARCKSL1, MYL12B, PKDCC, PRDX6, RHOBTB3, RPL26L1, SEMA7A, SRGN, TIMP3, TNC
	collagen-containing extracellular matrix	0.0464	1.3326	ADAMTSL4, COL12A1, HSPG2, IGFBP7, SEMA7A, TIMP3, TNC
	adherens junction	0.0472	1.3253	AHNAK, CAV2, FLNB, FZD1, HSPG2, LRP1, OXTR, TNC
KEGG Pathway	Proteoglycans in cancer	0.0146	1.8348	CAV2, FLNB, FZD1, HSPG2, RPS6KB2, TIMP3
Transcription Factor	Factor: MAZ; motif: GGGGAGGG	0.0074	2.1302	ADAMTSL4, AHNAK, BEX1, C1R, CAV2, CFD, COL12A1, CRIP1, EBNA1BP2, FAM43A, FGF5, FH, FLNB, FTSJ1, HES4, HSPB6, HSPG2, IGFBP7, IL17RA, ITGB1BP1, ITM2C, KLC, LMO4, LRP1, MARCKSL1, MRPL20, OSMR, PHLDA2, PKDCC, PLK3, PPP1R14A, PRDX6, PRRC2B, PRRC2C, RHOBTB3, RPS6KB2, RRP36, SELENOH, SELENOW, SEMA7A, SLC25A23, SOX4, TIMP3, WFS1
	Factor: MAZ; motif: GGGGAGGG; match class: 0	0.0074	2.1302	ADAMTSL4, AHNAK, BEX1, C1R, CAV2, CFD, COL12A1, CRIP1, EBNA1BP2, FAM43A, FGF5, FH, FLNB, HSPB6, HSPG2, IGFBP7, IL17RA, ITGB1BP1, ITM2C, KLC1, LMO4, LRP1, MARCKSL1, MRPL20, OSMR, PHLDA2, PPP1R14A, PRDX6, PRRC2B, PRRC2C, RHOBTB3, RPS6KB2, RRP36, SELENOH, SELENOW, SEMA7A, SLC25A23, SOX4, TIMP3

				FTSJ1<br>HES4	PKDCC<br>PLK3	WFS1
	Factor: CKROX; motif: SCCCTCCCC	0.0335	1.4748	AHNAK<br>BEX1<br>C1R<br>CAV2<br>CFD<br>COL12A1<br>CRIP1<br>EBNA1BP2<br>FAM43A<br>FLNB<br>FTSJ1<br>HES4<br>HSPB6	HSPG2<br>IL17RA<br>ITM2C<br>KLC1<br>LMO4<br>LRP1<br>MARCKSL1<br>MRPL20<br>OSMR<br>PHLDA2<br>PKDCC<br>PLK3	PPP1R14A<br>PRDX6<br>PRRC2B<br>RHOBTB3<br>RPS6KB2<br>RRP36<br>SELENOW<br>SEMA7A<br>SLC25A23<br>SOX4<br>TIMP3<br>WFS1
Transcription Factor	Factor: CKROX; motif: SCCCTCCCC; match class: 0	0.0335	1.4748	AHNAK<br>BEX1<br>C1R<br>CAV2<br>CFD<br>COL12A1<br>CRIP1<br>EBNA1BP2<br>FAM43A<br>FLNB<br>FTSJ1<br>HES4<br>HSPB6	HSPG2<br>IL17RA<br>ITM2C<br>KLC1<br>LMO4<br>LRP1<br>MARCKSL1<br>MRPL20<br>OSMR<br>PHLDA2<br>PKDCC<br>PLK3<br>PPP1R14A	PRDX6<br>PRRC2B<br>RHOBTB3<br>RPS6KB2<br>RRP36<br>SELENOW<br>SEMA7A<br>SLC25A23<br>SOX4<br>TIMP3<br>WFS1
	Factor: Egr-1; motif: GCGGGGGCGG; match class: 1	0.0382	1.4170	AHNAK<br>BEX1<br>CFD<br>COL12A1<br>FH<br>FLNB<br>FZD1<br>HES4	HSPG2<br>IL17RA<br>KLF2<br>LMO4<br>LRP1<br>MARCKSL1<br>MRPL20<br>PHLDA2	PKDCC<br>PRDX6<br>PRRC2B<br>RHOBTB3<br>SEMA7A<br>SLC25A23<br>TIMP3<br>WFS1

S5 Table . g:Profiler results from the analysis of 58 genes differentially expressed in four proband fibroblast lines cultured at normoxia compared to three control fibroblast lines cultured at normoxia. Analysis was performed using the default settings and selecting the Ensembl ID with the most annotations for each gene name. The p-value cut-off was 0.05 after g:SCS significance adjustment.
