# Supplementary material for: Disruption of the HIF-1 pathway in individuals with Ollier disease and Maffucci syndrome
Source: PLoS Genet. 2022 Dec 8;18(12):e1010504. doi: 10.1371/journal.pgen.1010504 (PMC9767349; doi:10.1371/journal.pgen.1010504)
Supplement: S6 Table — Analysis was performed using the default settings and selecting the Ensembl ID with the most annotations for each gene name. The p-value cut-off was 0.05 after g:SCS significance adjustment. (PDF) [file pgen.1010504.s008.pdf]

Full g:Profiler Results: Proband, Hypoxia (3) vs. Control, Hypoxia (3)

Source	*Term*	*Adj. P*	*-log$_{10}$ Adj. P*	*Sig. Genes in Term*		
GO: Biological Process	artery development	0.0002	3.5691	HES1<br>PKD2	PLXND1<br>SMAD7	TBX2
	heart morphogenesis	0.0013	2.8801	GAA<br>HES1	PKD2<br>PLXND1	SMAD7<br>TBX2
	aorta development	0.0014	2.8315	HES1<br>PKD2	PLXND1	TBX2
	gland development	0.0018	2.7425	HES1<br>LBH<br>PKD2	PLXND1<br>TBX2<br>TGM2	TNC
	tube morphogenesis	0.0019	2.7047	HES1<br>HOXA11<br>NR2F2	PKD2<br>PLXND1<br>SMAD7	TBX2<br>TGM2<br>TNC
	tissue morphogenesis	0.0021	2.6727	HES1<br>HOXA11<br>PKD2	PLXND1<br>SMAD7<br>TBX2	TGM2<br>TNC
	epithelium development	0.0033	2.4770	HES1<br>HOXA11<br>LBH<br>NR2F2	PKD2<br>PLXND1<br>SMAD7	TBX2<br>TGM2<br>TNC
	tissue development	0.0039	2.4043	GAA<br>GDF6<br>HES1<br>HOXA11	LBH<br>NR2F2<br>PKD2<br>PLXND1	SMAD7<br>TBX2<br>TGM2<br>TNC
	artery morphogenesis	0.0043	2.3576	HES1<br>PKD2	SMAD7	TBX2
	epithelial tube morphogenesis	0.0047	2.3266	HES1<br>HOXA11	PKD2<br>PLXND1	TBX2<br>TNC
	morphogenesis of a branching epithelium	0.0054	2.2672	HOXA11<br>PKD2	PLXND1<br>TGM2	TNC
	animal organ morphogenesis	0.0058	2.2320	GAA<br>HES1<br>HOXA11	PKD2<br>PLXND1<br>SMAD7	TBX2<br>TGM2<br>TNC
	morphogenesis of an epithelium	0.0062	2.2031	HES1<br>HOXA11<br>PKD2	PLXND1<br>TBX2	TGM2<br>TNC
	morphogenesis of a branching structure	0.0077	2.1105	HOXA11<br>PKD2	PLXND1<br>TGM2	TNC
	tube development	0.0095	2.0222	HES1<br>HOXA11<br>NR2F2	PKD2<br>PLXND1<br>SMAD7	TBX2<br>TGM2<br>TNC
	ureteric bud development	0.0121	1.916211	HES1<br>HOXA11	PKD2	SMAD7
	mesonephric epithelium development	0.0126	1.8986	HES1<br>HOXA11	PKD2	SMAD7
	mesonephric tubule development	0.0126	1.8986	HES1<br>HOXA11	PKD2	SMAD7
	mesonephros development	0.0147	1.8303	HES1<br>HOXA11	PKD2	SMAD7
	placenta blood vessel development	0.0172	1.7635	HES1	NR2F2	PKD2

GO: Biological Process	cardiac septum development	0.0206	1.6860	HES1<br>PLXND1	SMAD7	TBX2
	gland morphogenesis	0.0288	1.5392	PLXND1<br>TBX2	TGM2	TNC
	regulation of cell population proliferation	0.0413	1.3835	BEX4<br>FTH1<br>HES1<br>LBH	MAB21L1<br>NR2F2<br>PKD2	TBX2<br>TGM2<br>TNC
Human Protein Atlas	placenta; decidual cells [Approved, Medium]	0.0062	2.2039	C1ORF198<br>FTH1<br>GLIPR1<br>LBH	LDLRAD2<br>PKD2<br>PLXND1<br>PVR	SMAD7<br>TBX2<br>TNC
	caudate; glial cells. [Approved, Medium]	0.0259	1.5864	C1ORF198<br>FTH1<br>GLIPR1	LBH<br>PLXND1<br>PVR	SMAD7<br>TBX2

S6 Table. g:Profiler results from the analysis of 28 genes differentially expressed in three proband fibroblast lines cultured at hypoxia compared to three control fibroblast lines cultured at hypoxia. Analysis was performed using the default settings and selecting the Ensembl ID with the most annotations for each gene name. The p-value cut-off was 0.05 after g:SCS significance adjustment.
