# Supplementary material for: Disruption of the HIF-1 pathway in individuals with Ollier disease and Maffucci syndrome
Source: PLoS Genet. 2022 Dec 8;18(12):e1010504. doi: 10.1371/journal.pgen.1010504 (PMC9767349; doi:10.1371/journal.pgen.1010504)
Supplement: S7 Table — Analysis was performed using the default settings and selecting the Ensembl ID with the most annotations for each gene name. The p-value cut-off was 0.05 after g:SCS significance adjustment. If number of significant genes in g:Profiler term is greater than 50, n is shown; full lists are available upon request. (PDF) [file pgen.1010504.s009.pdf]

Full g:Profiler Results: Proband, Hypoxia (3) vs. Proband, Normoxia (3)

Source	*Term*	*Adj. P*	*-log$_{10}$ Adj. P*	*Sig. Genes in Term*		
GO: Molecular Function	identical protein binding	3.1073 E-06	5.5076	n = 66		
	catalytic activity	2.3171 E-05	4.6350	n = 155		
	enzyme binding	3.9685 E-05	4.4013	n = 75		
	protein binding	4.7884 E-05	4.3198	n = 275		
	monosaccharide binding	6.2656 E-05	4.2030	ALDOA<br>EGLN1<br>G6PD<br>GPI	GYS1<br>M6PR<br>OGFOD1<br>P4HA2	PFKL<br>PFKP<br>TALDO1
GO: Biological Process	glucose catabolic process	1.60 E-08	7.7947	ALDOA<br>ENO1<br>ENO2<br>GAPDH	GPI<br>PFKL<br>PFKP<br>PGAM1	PGK1<br>TP53<br>TPI1
	canonical glycolysis	1.73 E-08	7.7611	ALDOA<br>ENO1<br>ENO2<br>GAPDH	GPI<br>PFKL<br>PFKP	PGAM1<br>PGK1<br>TPI1
	glucose catabolic process to pyruvate	1.73 E-08	7.7611	ALDOA<br>ENO1<br>ENO2<br>GAPDH	GPI<br>PFKL<br>PFKP	PGAM1<br>PGK1<br>TPI1
	NADH regeneration	1.73 E-08	7.7611	ALDOA<br>ENO1<br>ENO2<br>GAPDH	GPI<br>PFKL<br>PFKP	PGAM1<br>PGK1<br>TPI1
	generation of precursor metabolites and energy	2.52 E-08	7.5989	ACADVL<br>AKR7A2<br>ALDOA<br>BNIP3<br>CS<br>DDIT4<br>ENO1<br>ENO2<br>G6PD<br>GAPDH<br>GNPDA1<br>GPI	GYS1<br>IDH1<br>LDHA<br>LOXL2<br>MYBBP1A<br>NDUFB4<br>NDUFB8<br>NQO1<br>P4HA2<br>PFKL<br>PFKP<br>PGAM1	PGAM4<br>PGD<br>PGK1<br>PPP1R3D<br>PPP1R3G<br>PYGB<br>STBD1<br>TALDO1<br>TKT<br>TP53<br>TPI1<br>TXNRD1
GO: Cellular Compartment	cytoplasmic part	3.06 E-14	13.5141	n = 248		
	cytoplasm	1.03 E-13	12.9886	n = 276		
	cytosol	2.29 E-10	9.6401	n = 152		
	intracellular	1.17 E-08	7.9327	n = 307		
	intracellular part	1.81 E-08	7.7418	n = 306		
KEGG Pathways	Biosynthesis of amino acids	1.8 2E-10	9.7405	ALDOA<br>CS<br>ENO1<br>ENO2<br>GAPDH<br>GOT2	IDH1<br>PFKL<br>PFKP<br>PGAM1<br>PGAM4	PGK1<br>PYCR3<br>TALDO1<br>TKT<br>TPI1

KEGG Pathways	Carbon metabolism	2.12 E-09	8.6736	ALDOA<br>CS<br>ENO1<br>ENO2<br>G6PD<br>GAPDH	GOT2<br>GPI<br>IDH1<br>PFKL<br>PFKP<br>PGAM1	PGAM4<br>PGD<br>PGK1<br>TALDO1<br>TKT<br>TPI1
	Glycolysis / Gluconeogenesis	1.02 E-07	6.9931	ALDH3A2<br>ALDOA<br>ENO1<br>ENO2<br>GAPDH	GPI<br>LDHA<br>PFKL<br>PFKP	PGAM1<br>PGAM4<br>PGK1<br>TPI1
	HIF-1 signaling pathway	1.5769 E-06	5.8022	ALDOA<br>EGLN1<br>ENO1<br>ENO2<br>GAPDH	LDHA<br>LTBR<br>PFKL<br>PGK1<br>SERPINE1	SLC2A1<br>TFRC<br>TIMP1<br>VEGFA
	Pentose phosphate pathway	1.1786 E-05	4.9286	ALDOA<br>G6PD<br>GPI	PFKL<br>PFKP<br>PGD	TALDO1<br>TKT
REACTOME Pathways	Metabolism of carbohydrates	2.3926 E-06	5.6211	ALDOA<br>CHP1<br>CHST3<br>ENO1<br>ENO2<br>G6PD<br>GAPDH<br>GNPDA1	GOT2<br>GPI<br>GYS1<br>HAS2<br>HYAL2<br>PFKL<br>PFKP<br>PGAM1	PGD<br>PGK1<br>PYGB<br>SLC2A1<br>TALDO1<br>TKT<br>TPI1
	Gluconeogenesis	4.203 E-06	5.3764	ALDOA<br>ENO1<br>ENO2	GAPDH<br>GOT2<br>GPI	PGAM1<br>PGK1<br>TPI1
	Glycolysis	4.1106 E-05	4.3860	ALDOA<br>ENO1<br>ENO2<br>GAPDH	GNPDA1<br>GPI<br>PFKL<br>PFKP	PGAM1<br>PGK1<br>TPI1
	Glucose metabolism	6.0413 E-05	4.2188	ALDOA<br>ENO1<br>ENO2<br>GAPDH	GNPDA1<br>GOT2<br>GPI<br>PFKL	PFKP<br>PGAM1<br>PGK1<br>TPI1
	Metabolism	0.0078	2.1062	n = 68		
Wiki Pathways	Metabolic reprogramming in colon cancer	3.58 E-12	11.4455	ENO1<br>FASN<br>G6PD<br>GAPDH<br>GOT2	GPI<br>LDHA<br>PFKL<br>PGAM1<br>PGD	PGK1<br>SLC16A3<br>SLC2A1<br>TALDO1<br>TKT
	Cori Cycle	6.54 E-11	10.1845	G6PD<br>GAPDH<br>GPI<br>LDHA	PFKP<br>PGAM1<br>PGK1	SLC2A1<br>TALDO1<br>TPI1
	Glycolysis and Gluconeogenesis	4.42 E-09	8.3546	ALDOA<br>ENO1<br>ENO2<br>GAPDH<br>GOT2	GPI<br>LDHA<br>PFKL<br>PFKP	PGAM1<br>PGK1<br>SLC2A1<br>TPI1
	Photodynamic therapy-induced HIF-1 survival signaling	1.7999 E-06	5.7447	BNIP3<br>BNIP3L<br>EGLN1<br>LDHA	PFKL<br>PGK1<br>SERPINE1	SLC2A1<br>TP53<br>VEGFA
	Pathways in clear cell renal cell carcinoma	2.2839 E-06	5.6413	ALDOA<br>ENO1<br>ENO2<br>FASN<br>GAPDH	GPI<br>LDHA<br>PFKL<br>PFKP<br>PGK1	SLC2A1<br>SQSTM1<br>TPI1<br>VEGFA

Transcription Factor	Factor: ZF5; motif: GSGCGCGR; match class: 1	2.87 E-19	18.5428	n = 326
	Factor: E2F-3:HES-7; motif: NNNSGCGCSNNNNNCRCGYGNN; match class: 1	2.24 E-17	16.6492	n = 334
	Factor: MAZ; motif: GGGMGGGGSSGGGGGGGGGGG; match class: 1	2.09 E-15	14.6808	n = 320
	Factor: E2F-1:HES-7; motif: GGCRCGTGSYNNWNGGCGCSM; match class: 1	2.24 E-14	13.6506	n = 333
	Factor: ZF5; motif: NRNGNGCGCGCWN; match class: 0	3.02 E-14	13.5201	n = 330
miRNA sites	hsa-miR-331-3p	2.671 E-06	5.5733	ACTG1 ANKRD52 APBB1 BTG1 CAD DDX21 FASN FCHSD1 FUT11 GAPDH GNB2 GPI HSPA8 HSPD1 KAT2A KDM6B LDHA MRPS26 MYBBP1A NCDN NOP2 PFKL PGAM1 POLR3H PSMD11 PSME3 TKT TPI1 UBQLN1 VARS
	hsa-miR-423-5p	5.1039 E-06	5.2920	ACTG1 ANKRD52 APEX1 CBX6 CLCN7 DDX21 ERRFI1 FAM98A FHOD1 GAPDH KDM6B MRPL34 MSANTD3 NDRG1 NOLC1 PGAM1 PGAM4 PNRC1 POLR3H PSMD11 RTL8C SAR1B SLC48A1 SQSTM1 TIMP3 TXNIP WDR45B
	hsa-miR-484	6.2184 E-05	4.2063	ADAR APEX1 ATP1A1 BTG1 C12ORF49 C1QBP CBX6 CCDC97 CLUH DDX41 DVL2 ENO1 FASN FUT11 GAPDH GCN1 GLTP GNB2 GOT2 GPATCH4 HSP90AB1 ITGA5 M6PR MDM2 NOLC1 PARP1 PFKL PGAM1 PGD POLR3H PSME3 PSMG1 PYGB RFX5 RRS1 SLC2A1 SQSTM1 SRPRA SRSF9 STIP1 TKT UBQLN1 USP5 VARS VKORC1
	hsa-let-7b-5p	0.0004	3.3827	N = 53
	hsa-miR-615-3p	0.01580	1.8011	ACADVL ALDOA BCKDK CBX6 HSPA8 IPO13 KAT2A KIAA1191 PGAM4 PLEKHB2 POLR3H SEMA7A

		DDIT4	NCL	SLC30A1
		DDX19A	NOLC1	SLC38A7
		ENO1	NOP2	TMEM126B
		FAM98A	NUDT16	TMSB10
		FASN	OGFOD1	TPI1
		GAPDH	PARP1	VARS
		HACD3	PCNX3	VDAC1
		HSP90AB1	PFKP	VIM
		HSPA4	PGAM1	VPS18

S7 Table. Top five g:Profiler results for each category from the analysis of 363 genes differentially expressed in three proband fibroblast lines cultured at hypoxia compared to the same three proband fibroblast lines cultured at normoxia. Analysis was performed using the default settings and selecting the Ensembl ID with the most annotations for each gene name. The p-value cut-off was 0.05 after g:SCS significance adjustment. If number of significant genes in g:Profiler term is greater than 50, *n* is shown; full lists are available upon request.
