# Supplementary material for: Disruption of the HIF-1 pathway in individuals with Ollier disease and Maffucci syndrome
Source: PLoS Genet. 2022 Dec 8;18(12):e1010504. doi: 10.1371/journal.pgen.1010504 (PMC9767349; doi:10.1371/journal.pgen.1010504)
Supplement: S8 Table — Analysis was performed using the default settings and selecting the Ensembl ID with the most annotations for each gene name. The p-value cut-off was 0.05 after g:SCS significance adjustment. If number of significant genes in g:Profiler term is greater than 50, n is shown; full lists are available upon request. (PDF) [file pgen.1010504.s010.pdf]

Full g:Profiler Results: Control, Hypoxia (3) vs. Control, Normoxia (3)

Source	*Term*	*Adj. P*	*-log$_{10}$ Adj. P*	*Sig. Genes in Term*		
GO: Molecular Function	monosaccharide binding	2.74 E-07	6.5624	ALDOA<br>EGLN1<br>GPI<br>GYS1	LMAN1<br>P4HA2<br>PFKL	PFKP<br>PLOD2<br>TALDO1
	intramolecular oxidoreductase activity, interconverting aldoses and ketoses	0.0003	3.5110	GNPDA1<br>GPI	MPI	TPI1
	carbohydrate binding	0.0012	2.8872	ALDOA<br>EGLN1<br>GPI<br>GYS1	LMAN1<br>P4HA2<br>PFKL<br>PFKP	PLOD2<br>PPP1R3G<br>STBD1<br>TALDO1
	oxidoreductase activity	0.0013	2.8668	ADH5<br>ALKBH5<br>APEX1<br>EGLN1<br>ERO1A<br>FTH1<br>GAPDH	HSD17B10<br>KIAA1191<br>LDHA<br>LOX<br>NQO1<br>P4HA2<br>P4HB	PGK1<br>PLOD2<br>RSAD1<br>SRXN1<br>TXNRD1<br>VKORC1
	intramolecular oxidoreductase activity	0.0015	2.8113	ERO1A<br>GNPDA1	GPI<br>MPI	P4HB<br>TPI1
GO: Biological Process	glucose catabolic process to pyruvate	4.17 E-14	13.3793	ALDOA<br>ALDOC<br>ENO1<br>ENO2	GAPDH<br>GPI<br>PFKL<br>PFKP	PGAM1<br>PGK1<br>TPI1
	canonical glycolysis	4.17 E-14	13.3793	ALDOA<br>ALDOC<br>ENO1<br>ENO2	GAPDH<br>GPI<br>PFKL<br>PFKP	PGAM1<br>PGK1<br>TPI1
	NADH regeneration	4.17 E-14	13.3793	ALDOA<br>ALDOC<br>ENO1<br>ENO2	GAPDH<br>GPI<br>PFKL<br>PFKP	PGAM1<br>PGK1<br>TPI1
	glycolytic process through fructose-6-phosphate	7.19 E-14	13.1434	ALDOA<br>ALDOC<br>ENO1<br>ENO2	GAPDH<br>GPI<br>PFKL<br>PFKP	PGAM1<br>PGK1<br>TPI1
	glycolytic process through glucose-6-phosphate	7.19 E-14	13.1434	ALDOA<br>ALDOC<br>ENO1<br>ENO2	GAPDH<br>GPI<br>PFKL<br>PFKP	PGAM1<br>PGK1<br>TPI1
GO: Cellular Compartment	cytoplasmic part	4.64 E-06	5.3334	N=107		
	cytoplasm	6.67 E-06	5.17577	N=119		
	cytosol	1.85 E-05	4.7333	N=69		
	extracellular exosome	0.0001	3.8590	ADH5<br>ALDOA<br>ALDOC<br>CCT6A<br>CNDP2<br>ENO1<br>ENO2<br>FTH1<br>GAPDH	LDHA<br>LMAN1<br>MARCKSL1<br>MPI<br>MUC1<br>NDRG1<br>NEU1<br>P4HB<br>PFKL	PGM1<br>PLOD2<br>PTPRF<br>RAB29<br>SERPINE1<br>SLC2A1<br>SLC3A2<br>SQSTM1<br>TALDO1

				GNPDA1<br>GPI<br>HSPD1<br>HSPH1	PFKP<br>PGAM1<br>PGAM4<br>PGK1	TPI1<br>TXNRD1<br>VWA1
	extracellular vesicle	0.0001	3.7423	ADH5<br>ALDOA<br>ALDOC<br>CCT6A<br>CNDP2<br>ENO1<br>ENO2<br>FTH1<br>GAPDH<br>GNPDA1<br>GPI<br>HSPD1<br>HSPH1	LDHA<br>LMAN1<br>MARCKSL1<br>MPI<br>MUC1<br>NDRG1<br>NEU1<br>P4HB<br>PFKL<br>PFKP<br>PGAM1<br>PGAM4<br>PGK1	PGM1<br>PLOD2<br>PTPRF<br>RAB29<br>SERPINE1<br>SLC2A1<br>SLC3A2<br>SQSTM1<br>TALDO1<br>TPI1<br>TXNRD1<br>VWA1
KEGG Pathways	Glycolysis / Gluconeogenesis	6.99 E-15	14.1556	ADH5<br>ALDOA<br>ALDOC<br>ENO1<br>ENO2	GAPDH<br>GPI<br>LDHA<br>PFKL<br>PFKP	PGAM1<br>PGAM4<br>PGK1<br>PGM1<br>TPI1
	Carbon metabolism	5.20 E-10	9.2842	ADH5<br>ALDOA<br>ALDOC<br>ENO1<br>ENO2	GAPDH<br>GPI<br>PFKL<br>PFKP<br>PGAM1	PGAM4<br>PGK1<br>TALDO1<br>TPI1
	Biosynthesis of amino acids	6.23 E-10	9.2058	ALDOA<br>ALDOC<br>ENO1<br>ENO2	GAPDH<br>PFKL<br>PFKP<br>PGAM1	PGAM4<br>PGK1<br>TALDO1<br>TPI1
	HIF-1 signaling pathway	3.13 E-07	6.5042	ALDOA<br>EGLN1<br>ENO1<br>ENO2	GAPDH<br>LDHA<br>PFKL<br>PGK1	SERPINE1<br>SLC2A1<br>VEGFA
	Pentose phosphate pathway	1.15 E-06	5.9405	ALDOA<br>ALDOC<br>GPI	PFKL<br>PFKP	PGM1<br>TALDO1
REACTOME Pathways	Glycolysis	4.51 E-10	9.3455	ALDOA<br>ALDOC<br>ENO1<br>ENO2	GAPDH<br>GNPDA1<br>GPI<br>PFKL	PFKP<br>PGAM1<br>PGK1<br>TPI1
	Metabolism of carbohydrates	2.39 E-09	8.6215	ALDOA<br>ALDOC<br>CHST2<br>ENO1<br>ENO2<br>GAPDH<br>GNPDA1	GPI<br>GYS1<br>HAS2<br>PFKL<br>PFKP<br>PGAM1	PGK1<br>PGM1<br>SLC2A1<br>TALDO1<br>TPI1<br>XYLT2
	Gluconeogenesis	4.18 E-09	8.3785	ALDOA<br>ALDOC<br>ENO1	ENO2<br>GAPDH<br>GPI	PGAM1<br>PGK1<br>TPI1
	Glucose metabolism	8.59 E-09	8.0658	ALDOA<br>ALDOC<br>ENO1<br>ENO2	GAPDH<br>GNPDA1<br>GPI<br>PFKL	PFKP<br>PGAM1<br>PGK1<br>TPI1
	Metabolism	3.28 E-05	4.48433	ADH5<br>AIMP2<br>ALDOA<br>ALDOC<br>ANGPTL4<br>BCKDK<br>CA12<br>CACNB3<br>CHST2	GNPDA1<br>GPI<br>GYS1<br>HAS2<br>HILPDA<br>HSD17B10<br>INSIG2<br>LARS<br>LDHA	PFKP<br>PGAM1<br>PGK1<br>PGM1<br>PSMC2<br>SEPHS2<br>SLC16A3<br>SLC2A1<br>SLC3A2

				CNDP2<br>COX14<br>ENO1<br>ENO2<br>GAPDH<br>GLTP	NEU1<br>NQO1<br>ODC1<br>PCYT2<br>PFKL	TALDO1<br>TPI1<br>TXNRD1<br>VKORC1<br>XYLT2
Wiki Pathways	Pathways in clear cell renal cell carcinoma	1.01 E-13	12.9958	ALDOA<br>ALDOC<br>ENO1<br>ENO2<br>GAPDH<br>GPI	LDHA<br>PFKL<br>PFKP<br>PGK1<br>PGM1	PLOD2<br>SLC2A1<br>SQSTM1<br>TPI1<br>VEGFA
	Glycolysis and Gluconeogenesis	1.48 E-13	12.8290	ALDOA<br>ALDOC<br>ENO1<br>ENO2<br>GAPDH	GPI<br>LDHA<br>PFKL<br>PFKP	PGAM1<br>PGK1<br>SLC2A1<br>TPI1
	Cori Cycle	2.81 E-12	11.5512	GAPDH<br>GPI<br>LDHA	PFKP<br>PGAM1<br>PGK1	SLC2A1<br>TALDO1<br>TPI1
	Metabolic reprogramming in colon cancer	2.96 E-09	8.5287	ENO1<br>GAPDH<br>GPI<br>LDHA	PFKL<br>PGAM1<br>PGK1	SLC16A3<br>SLC2A1<br>TALDO1
	Photodynamic therapy-induced HIF-1 survival signaling	2.51 E-08	7.6000	BNIP3<br>BNIP3L<br>EGLN1	LDHA<br>PFKL<br>PGK1	SERPINE1<br>SLC2A1<br>VEGFA
Transcription Factor	Factor: Churchill; motif: CGGGNN	3.51 E-05	4.4548	N=135		
	Factor: Churchill; motif: CGGGNN; match class: 0	3.51 E-05	4.4548	N=135		
	Factor: HIF1A; motif: NNACGTGCNN; match class: 0	3.81 E-05	4.4195	ALDOA<br>BCKDK<br>BNIP3<br>BNIP3L<br>C11ORF98<br>C4ORF3<br>CA12<br>CACNB3<br>CCT6A<br>DDX21<br>DDX41<br>ENO1<br>ENO2<br>ERO1A<br>FUT11	GAPDH<br>HAS2<br>HILPDA<br>KCTD11<br>LDHA<br>MAPK7<br>METTL26<br>MOSPD3<br>NAB2<br>NOP56<br>NPM3<br>OSMR<br>P4HA2<br>PFKL<br>PFKP	PLXNA3<br>PPP1R3G<br>PRMT6<br>PTPRF<br>RSAD1<br>SEPHS2<br>SLC16A3<br>SMIM3<br>STAT1<br>STC1<br>TPI1<br>VEGFA<br>VKORC1<br>WDR54
	Factor: HIF1A; motif: NNACGTGCNN	3.81 E-05	4.4195	ALDOA<br>BCKDK<br>BNIP3<br>BNIP3L<br>C11ORF98<br>C4ORF3<br>CA12<br>CACNB3<br>CCT6A<br>DDX21<br>DDX41<br>ENO1<br>ENO2<br>ERO1A<br>FUT11	GAPDH<br>HAS2<br>HILPDA<br>KCTD11<br>LDHA<br>MAPK7<br>METTL26<br>MOSPD3<br>NAB2<br>NOP56<br>NPM3<br>OSMR<br>P4HA2<br>PFKL<br>PFKP	PLXNA3<br>PPP1R3G<br>PRMT6<br>PTPRF<br>RSAD1<br>SEPHS2<br>SLC16A3<br>SMIM3<br>STAT1<br>STC1<br>TPI1<br>VEGFA<br>VKORC1<br>WDR54

	Factor: HIF1; motif: GNNKACGTGCGGNN; match class: 0	4.17 E-05	4.3796	n = 56
miRNA Sites	hsa-miR-331-3p	0.0182	1.7394	APBB1<br>APOL6<br>DDX21<br>FUT11<br>GAPDH<br>GPI<br>HSPD1<br>LARS<br>LDHA<br>MARCKSL1<br>NOP2<br>PFKL<br>PGAM1<br>TPI1
Human Protein Atlas	urinary bladder; urothelial cells [Approved, Low]	0.0313	1.5042	n = 62

S8 Table. Top five g:Profiler results for each category from the analysis of 154 genes differentially expressed in three control fibroblast lines cultured at hypoxia compared to the same three control fibroblast lines cultured at normoxia. Analysis was performed using the default settings and selecting the Ensembl ID with the most annotations for each gene name. The p-value cut-off was 0.05 after g:SCS significance adjustment. If number of significant genes in g:Profiler term is greater than 50, *n* is shown; full lists are available upon request.
