# Supplementary material for: Disruption of the HIF-1 pathway in individuals with Ollier disease and Maffucci syndrome
Source: PLoS Genet. 2022 Dec 8;18(12):e1010504. doi: 10.1371/journal.pgen.1010504 (PMC9767349; doi:10.1371/journal.pgen.1010504)
Supplement: S9 Table — (PDF) [file pgen.1010504.s011.pdf]

| Proband ID | Gene mutated | Variant | Forward Primer | Reverse Primer |
|---|---|---|---|---|
| 1 | *HIF1A* | p.Val74Leu | ggaaacacctgcttccgaca | gggaggggaaaagccagtat |
| 2 | *HIF1A* | p.Pro239Leu | ctgggcaggaagtaggtcat | ggctgtgtcgactgaggaaa |
| 3 | *HIF1A* | p.Asp446Tyr | gtgaaaatccttgtggccgg | tccatcggaaggactaggtgt |
| 4 | *HIF1A* | p.Arg655His | aaagcgcaagtcctcaaagc | acacgttagggcttcttgga |
| 5 | *HIF1A* | p.Ala678Val | ccactgccaccactgatgaa | tgagatggtgccatgccatt |
| 6 | *HIF1A* | p.Ser716Cys | actagtgccacatcatcacca | tgagatggtgccatgccatt |
| 6 | *IDH1* | p.Arg132His | atgtgttgagatggacgcct | atattctgggtggcacggtc |
| 7 | *HIF1A* | p.Glu481Lys | ccccacagacacagaaactga | atcctgaatctggggcatgg |
| 7 | *VHL* | p.Pro25Leu | gttccatcctctaccgagcg | gcagaagatgacctgggagg |
| 8 | *VHL* | p.Pro25Leu | gttccatcctctaccgagcg | gcagaagatgacctgggagg |
| 9 | *VHL* | p.Glu52Lys | cgaagactacggaggtcgac | tcgaagttgagccatacggg |
| 10 | *VHL* | p.Pro81Ser | gtacggccctgaagaagacg | gcttcagaccgtgctatcgt |
| 11 | *VHL* | p.Ile180Val | caagcccagcccatttcaag | tcccatccgttgatgtgcaa |
| 12 | *VHL* | p.Arg210Trp | gactggacatcgtcaggtcg | aatgccaccaccttctcctg |
| 13 | *IDH1* | p.Arg132His | atgtgttgagatggacgcct | atattctgggtggcacggtc |
| 14 | *IDH1* | p.Ile189Val | taactgggactagaggcgcc | gttggaaacctgtctgggact |
| 16 | *IDH2* | p.Thr435Met | tggctgaggtaaaacgcact | atggctgttctgatgcccaa |
| 17 | *IDH2* | p.Asp225Asn | agatggggtctcactgttgc | gccggcactttcaaaatggt |
| 18 | *KDM4C* | p.Tyr4Cys | cctcactccacagatggctg | ctgagcctgggatgttgagg |
| 19 | *KDM4C* | p.Arg371Gln | tccaacccggttgtgtttca | tgccatgtcgagcaacttca |
| 19 | *CDKN2A* | p.Ala121Thr | atggttactgcctctggtgc | gtgagggggctctacacaag |

**S9 Table.** Primer pairs designed for genomic DNA amplification and Sanger sequencing of candidate variants.
